# Supplementary material for: C2-Symmetric P-Stereogenic Ferrocene Ligands with Heavier Chalcogenophosphinous Acid Ester Donor Sites
Source: Molecules. 2021 Mar 27;26(7):1899. doi: 10.3390/molecules26071899 (PMC8037359; doi:10.3390/molecules26071899)
Supplement: Supplementary file 1 [file molecules-26-01899-s001.pdf]

Supplementary Material for:

C_2-Symmetric P-chiral ferrocene ligands with heavier chalcogenophosphinous acid ester donor sites

Roman Franz, Clemens Bruhn and Rudolf Pietschnig*

Institute for Chemistry and CINSaT, University of Kassel, Heinrich Plett-Straße 40, 34132 Kassel, Germany;

pietschnig@uni-kassel.de

Content:

a) X-ray crystallography
b) NMR spectra

X-ray crystallography

Table S1: Summary of structure determinations and refinement for **3a**, **3b**, **3c** and **4a**.

	**3a**	**3b**	**3c**	**4a**
CCDC code	2069749	2069744	2069748	2069746
Empirical formula	$C_{30}H_{36}FeP_2S_2$	$C_{30}H_{36}FeP_2Se_2$	$C_{30}H_{36}FeP_2Te_2$	$C_{20}H_{32}FeP_2S_2$
Formula weight [g/mol]	578.50	672.30	769.58	454.36
Crystal description	yellow needle	yellow block	yellow plate	yellow plate
Temperature [K]	100(2)	100(2)	100(2)	100(2)
Radiation and λ [Å]	Cu K_{α}, 1.54186	Mo K_{α}, 0.71073	Cu K_{α}, 1.54186	Mo K_{α}, 0.71073
Crystal system, space	monoclinic, $P2_1$/c	trikilinic, $P\bar{1}$	trikilinic, $P\bar{1}$	orthorhombic, *Pbcn*
Unit cell dimensions:				
a [Å]	5.9329(2)	10.3745(6)	9.6494(6)	17.0861(8)
b [Å]	26.6620(9)	10.9229(7)	10.5509(7)	11.2998(4)
c [Å]	26.8428(8)	14.1258(8)	15.8127(10)	11.6829(4)
α [°]	90	105.876(4)	108.752(5)	90
β [°]	91.348(2)	99.149(4)	90.726(5)	90
γ [°]	90	105.170(5)	101.888(5)	90
Volume [Å^3]	4244.9(2)	1439.45(16)	1486.32(17)	2255.61(15)
Z	6	2	2	4
Crystal size [mm]	0.26x0.11x0.03	0.16x0.13x0.11	0.23x0.15x0.04	0.13 x 0.08x0.05
Calculated density [g/cm^3]	1.358	1.551	1.720	1.338
Linear absorption coefficient μ [mm^{-1}]	6.844	3.181	20.383	0.997
F(000)	1824	680	752	960
Θ-Range for data collection [°]	3.32 -70.88	1.55-26.83	2.96 - 70.76	2.16-25.61
Index ranges	$-7 < h < 3$	$-13 < h < 13$	$-11 < h < 8$	$-20 < h < 20$
	$-32 < k < 20$	$-13 < k < 13$	$-12 < k < 10$	$-13 < k < 13$
	$-28 < l < 32$	$-17 < l < 17$	$-18 < l < 19$	$-12 < l < 14$
Refl. collected/unique	15405/ 7755	11459/6077	10230/5370	12536/2128
Completeness to Θ = 26.0°	0.947	0.983	0.938	0.997
Data/restraints/parameters	7755/0/484	6077/0/322	5370/0/323	2128/0/118
Goodness-of-fit on F^2	1.037	1.038	1.154	1.051
Final *R* indices [I>2$\delta(I)$]/ [wR_2]	0.0325/0.0756	0.0428/0.1097	0.0921/0.2656	0.0482/0.1278
R indices (all data)/ [wR_2]	0.0416/0.0799	0.0506/0.1158	0.0984/0.2783	0.0550/0.1326
Largest difference hole/peak [e Å^{-3}]	-0.33, 0.74	-0.82, 2.41	-2.22, 2.35	-0.35, 1.62

Table S2: Summary of structure determinations and refinement for **4b**, **5** and **6**.

	**4b**	**5**	**6**
CCDC code	2069752	2069745	2069750
Empirical formula	$C_{20}H_{32}FeP_2Se_2$	$C_{32}H_{39}BCuF_4FeNP_2Se_2$	$C_{32}H_{39}AgBF_4FeNP_2Se_2$
Formula weight [g/mol]	548.16	863.70	908.03
Crystal description	yellow plate	yellow plate	yellow plate
Temperature [K]	100(2)	100(2)	100(2)
Radiation and λ [Å]	Cu K_α, 1.54186	Mo K_α, 0.71073	Cu K_α, 1.54186
Crystal system, space	orthorhombic, *Pbcn*	triclinic, $P\bar{1}$	triclinic, $P\bar{1}$
Unit cell dimensions:			
a [Å]	17.0990(8)	10.3974(4)	10.4916(7)
b [Å]	11.6204(5)	16.6880(8)	16.3942(8)
c [Å]	11.7868(7)	20.2031(8)	20.6054(13)
α [°]	90	81.760(4)	81.671(5)
β [°]	90	83.161(3)	83.251(5)
γ [°]	90	79.987(4)	81.309(5)
Volume [$Å^3$]	2342.0(2)	3400.5(3)	3449.9(4)
Z	4	4	4
Crystal size [mm]	0.09x0.067x 0.03	0.10x0.07x0.03	0.13x0.08x0.03
Calculated density [g/cm^3]	1.555	1.746	1.748
Linear absorption coefficient μ [mm^{-1}]	10.005	3.333	11.603
F(000)	1104	1728	1800
Θ-Range for data collection [°]	4.60-70.91	1.25-26.83	2.75-71.01
Index ranges	$-17 < h < 20$	$-13 < h < 13$	$-19 < h < 19$
	$-6 < k < 14$	$-21 < k < 21$	$-25 < k < 25$
	$-13 < l < 14$	$-25 < l < 25$	$-12 < l < 25$
Refl. collected/unique	6072/2215	26529/14343	25449/12530
Completeness to Θ = 26.0°	0.977	0.983	0.940
Data/restraints/parameters	2215/0/126	14343/0/807	12530/0/807
Goodness-of-fit on F^2	1.039	1.029	1.035
Final *R* indices [$I>2\delta(I)$]/ [wR_2]	0.0607/0.1471	0.0462/0.1067	0.0251/0.0615
R indices (all data)/ [wR_2]	0.0828/0.1630	0.0772/0.1301	0.0296/0.0638
Largest difference hole/peak [e $Å^{-3}$]	-0.74, 0.67	-0.92/1.11	-0.44/0.70

Table S3: Summary of structure determinations and refinement for **7** and **8**.

	**7**	**8**
CCDC code	2069751	2069747
Empirical formula	$C_{30}H_{36}Au_2Cl_2FeP_2Se_2$	C60 H72 Au2 Fe2 P4 Se4, 2(Al Cl4), 2(C6 H4 F2)
Formula weight [g/mol]	1137.13	2304.27
Crystal description	yellow plate	yellow plate
Temperature [K]	100(2)	100(2)
Radiation and λ [Å]	Cu K_{α}, 1.54186	Mo K_{α}, 0.71073
Crystal system, space	monoclinic, $P2_1/c$	triclinic, $P\bar{1}$
Unit cell dimensions:		
a [Å]	16.7987(6)	10.6470(4)
b [Å]	8.9299(2)	15.6378(7)
c [Å]	21.9642(7)	26.3155(13)
α [°]	90	89.832(4)
β [°]	90.199(3)	96.969(4)
γ [°]	90	109.470(3)
Volume [Å^3]	3294.85(17)	4097.1(3)
Z	4	2
Crystal size [mm]	0.09x0.06x0.04	0.22x0.147x0.04
Calculated density [g/cm^3]	2.292	1.868
Linear absorption coefficient μ [mm^{-1}]	24.980	6.103
F(000)	2128	2232
Θ-Range for data collection [°]	3.31 -71.6	1.38 - 25.70
Index ranges	$-19 < h < 20$	$-12 < h < 12$
	$-10 < k < 5$	$-18 < k < 19$
	$-26 < l < 23$	$-31 < l < 32$
Refl. collected/unique	13217/6062	28725/1532
Completeness to Θ = 26.0°	0.959	0.985
Data/restraints/parameters	6062/3/358	1532/204/955
Goodness-of-fit on F^2	1.108	1.055
Final R indices [I>2δ(I)]/ [wR_2]	0.0213/0.0495	0.0471/0.1177
R indices (all data)/ [wR_2]	0.0245/0.0510	0.0606/0.1365
Largest difference hole/peak [e Å^{-3}]	-0.89/1.14	-2.23/1.83

On the following pages the NMR-spectra of compounds **3a**, **3b**, **3c**, **4a**, **4b**, **5**, **6**, **7** and **8** are depicted:

NMR-spectra of **3a**:

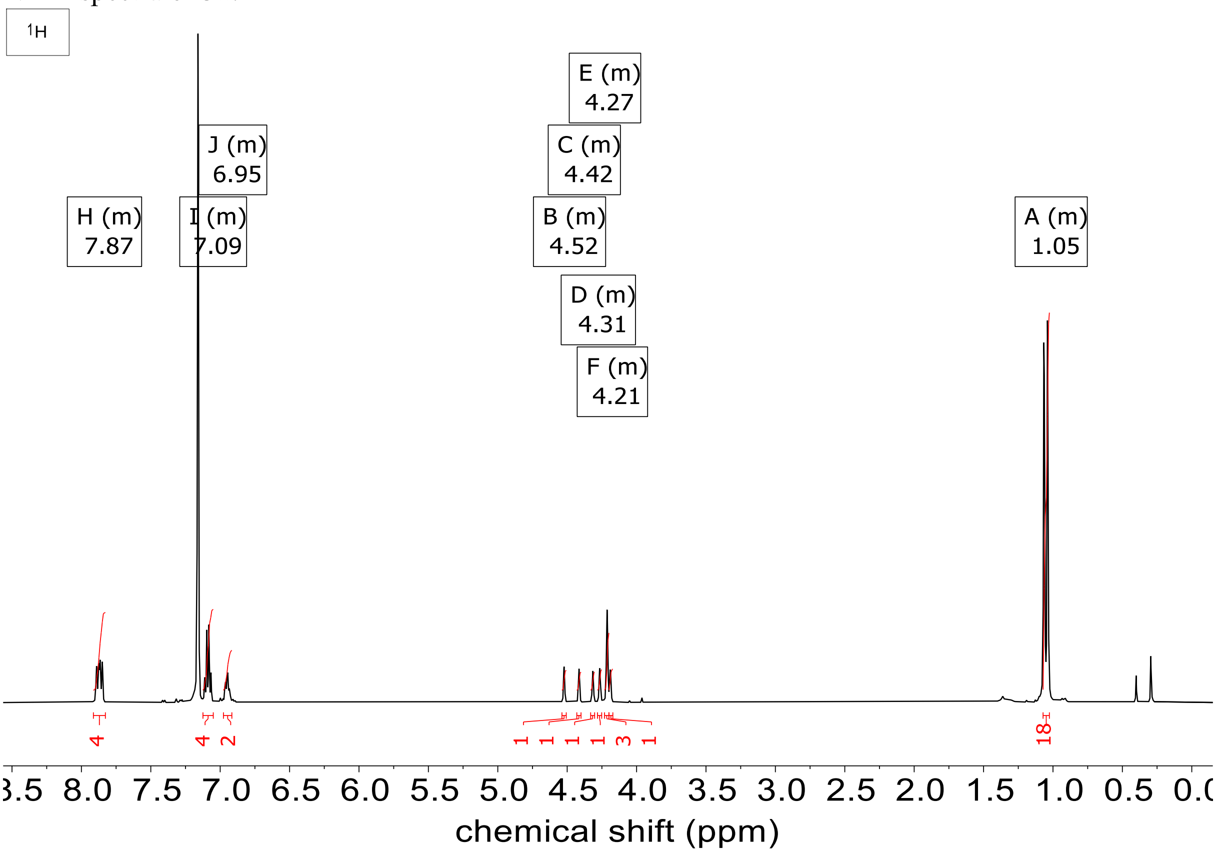

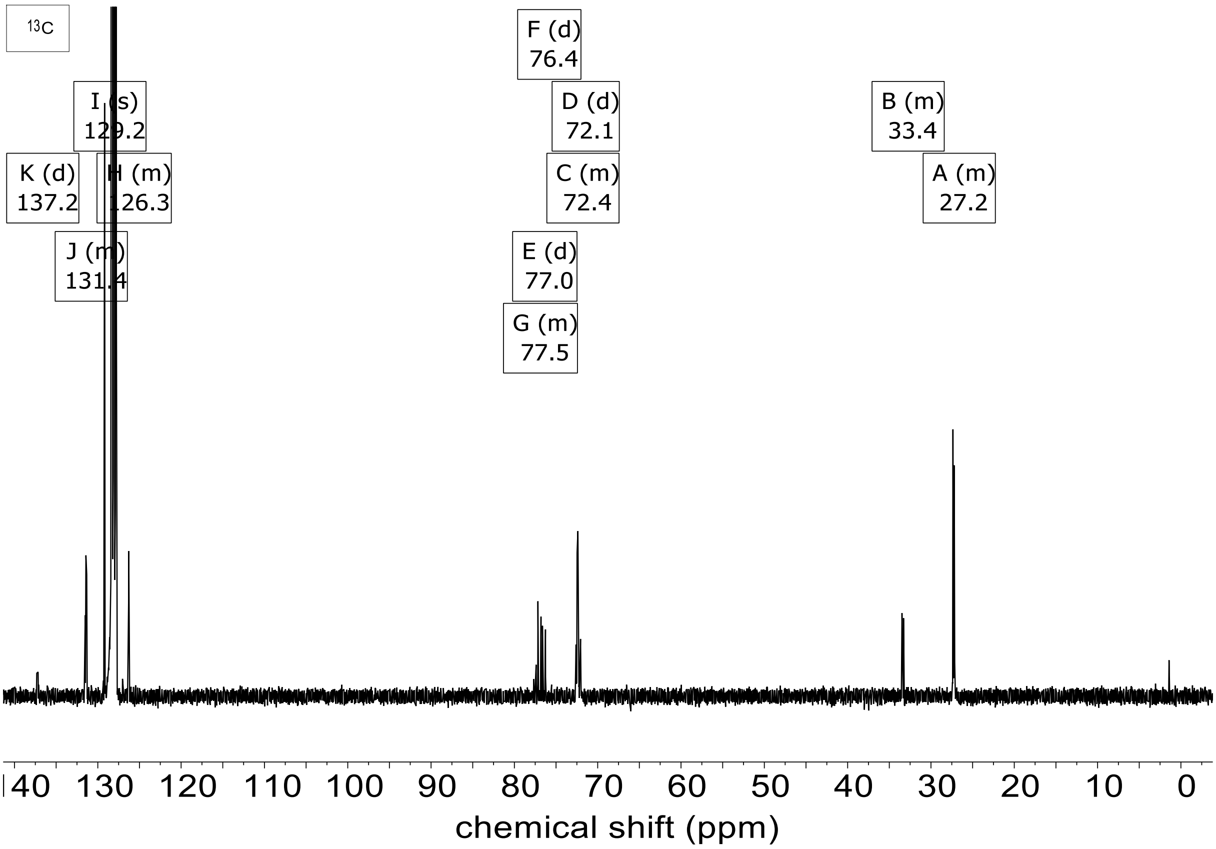

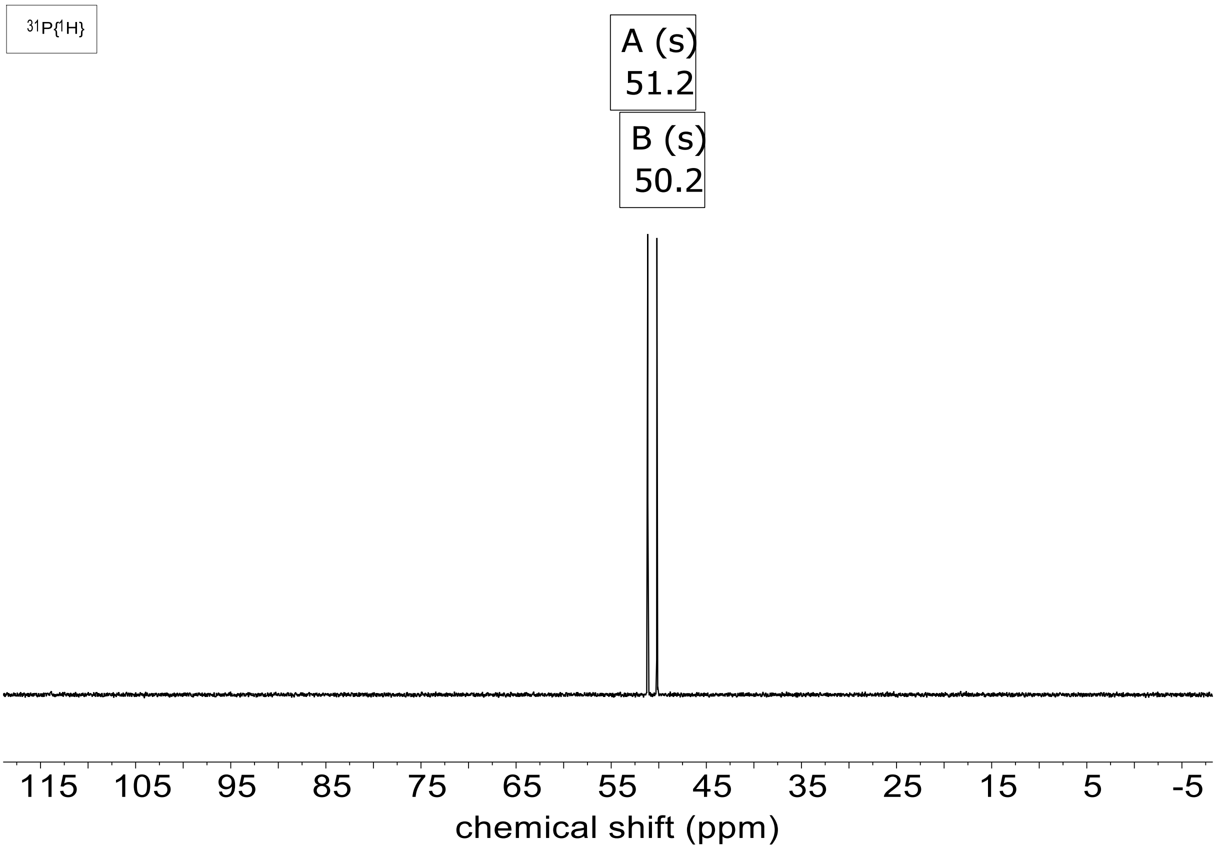

NMR-spectra of **3b**:

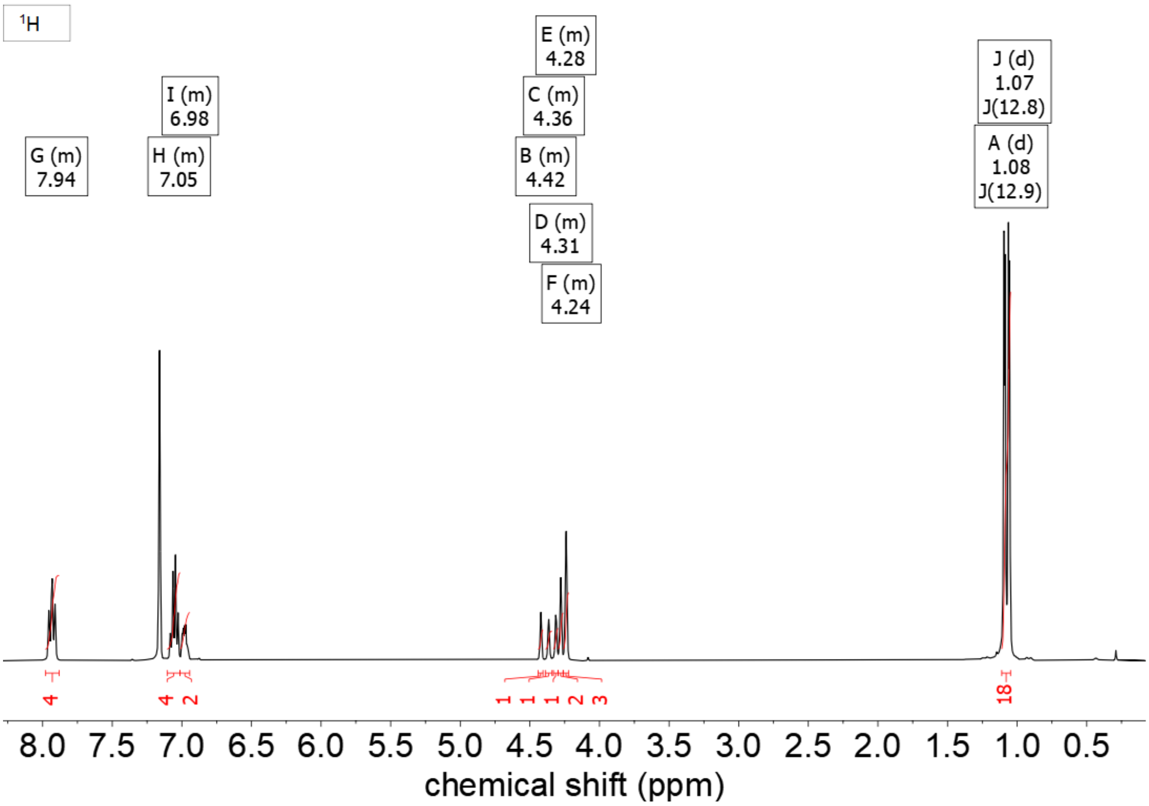

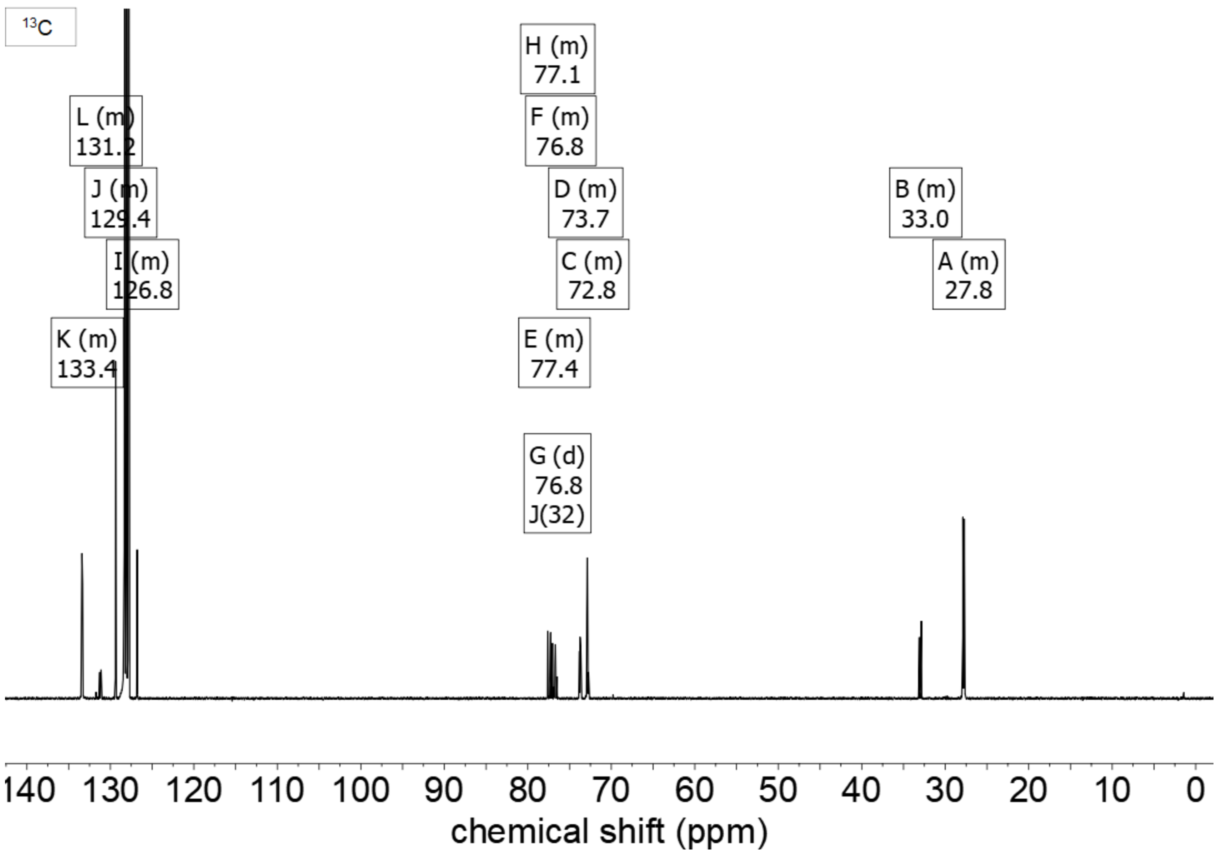
13C
H (m) 77.1
L (m) 131.2
F (m) 76.8
J (m) 129.4
D (m) 73.7
B (m) 33.0
I (m) 126.8
C (m) 72.8
A (m) 27.8
K (m) 133.4
E (m) 77.4
G (d) 76.8 J(32)
140 130 120 110 100 90 80 70 60 50 40 30 20 10 0
chemical shift (ppm)

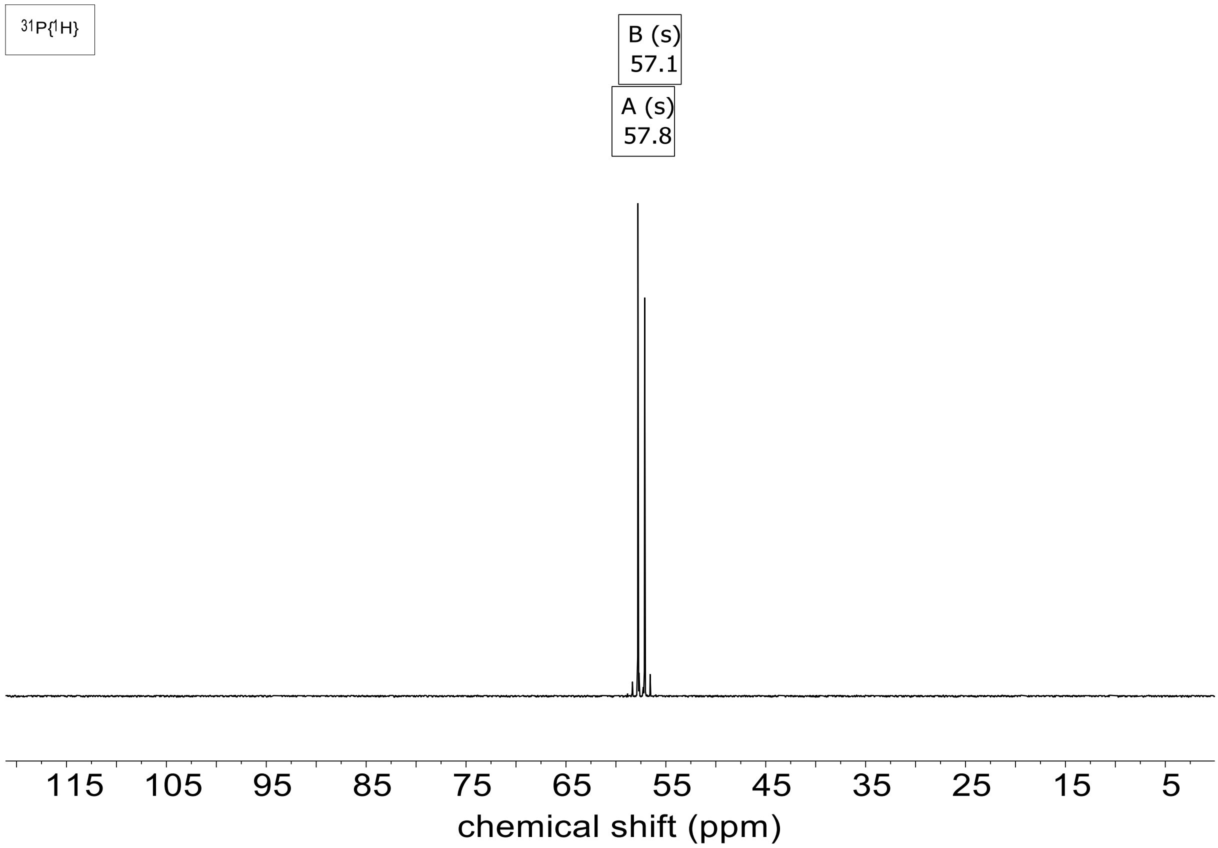
31P{1H}
B (s) 57.1
A (s) 57.8
115 105 95 85 75 65 55 45 35 25 15 5
chemical shift (ppm)

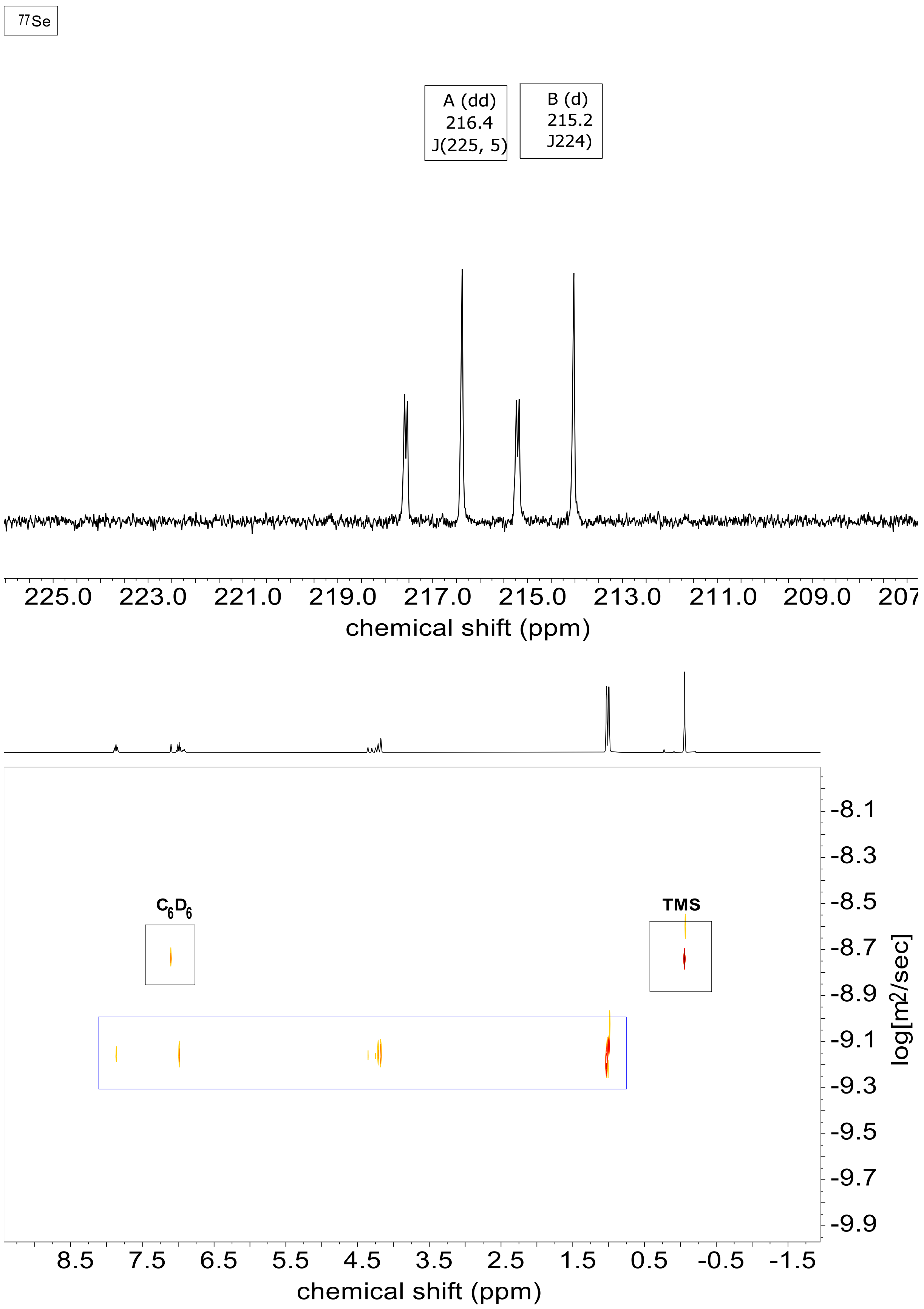

Figure S1: DOSY-nmr in benzene-*d6* as solvent and tetramethylsilane (TMS) as internal standard for calibration. $\mathbf{ECC^{Merge}_{C_6D_6}}$ was used to determine $MW_{det,\ cor}$ = 700 g/mol ($MW_{dif,\ cor}$ = -4%).

NMR-spectra of **3c**:

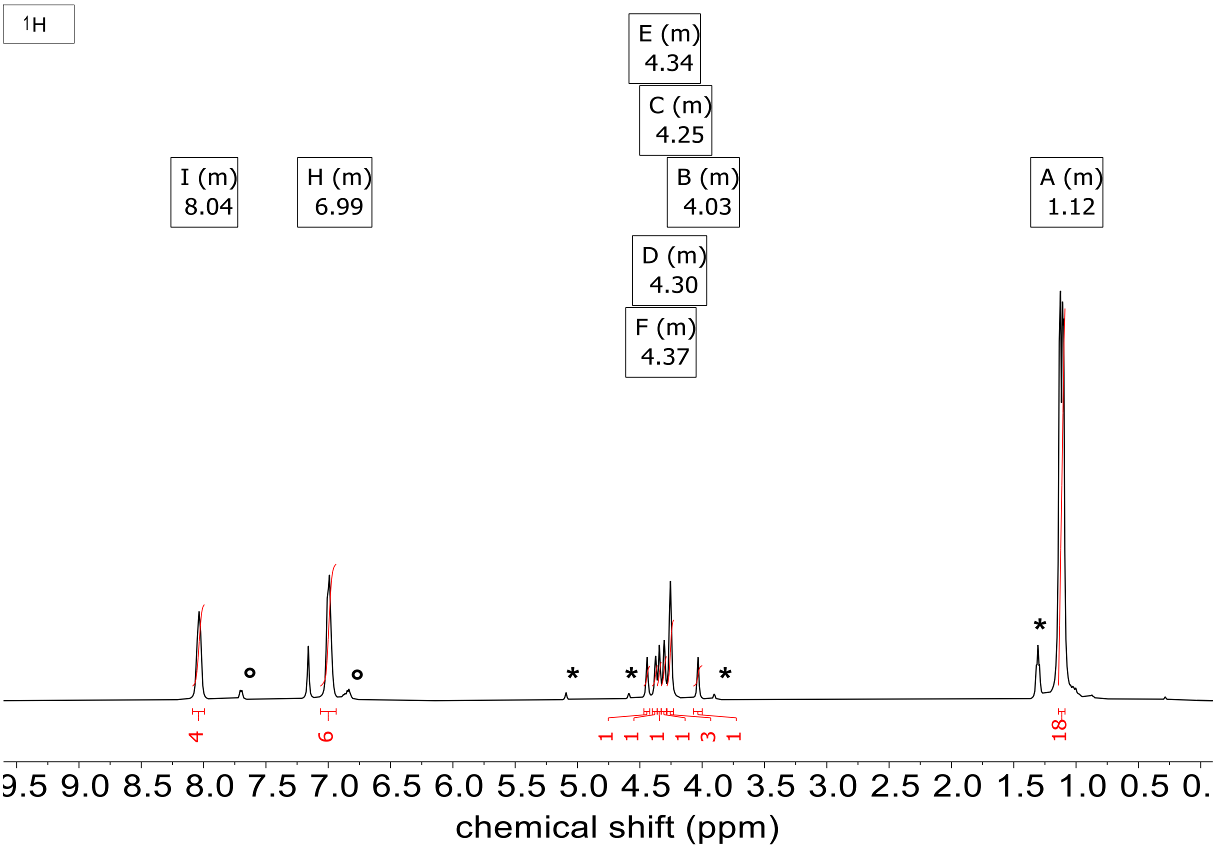

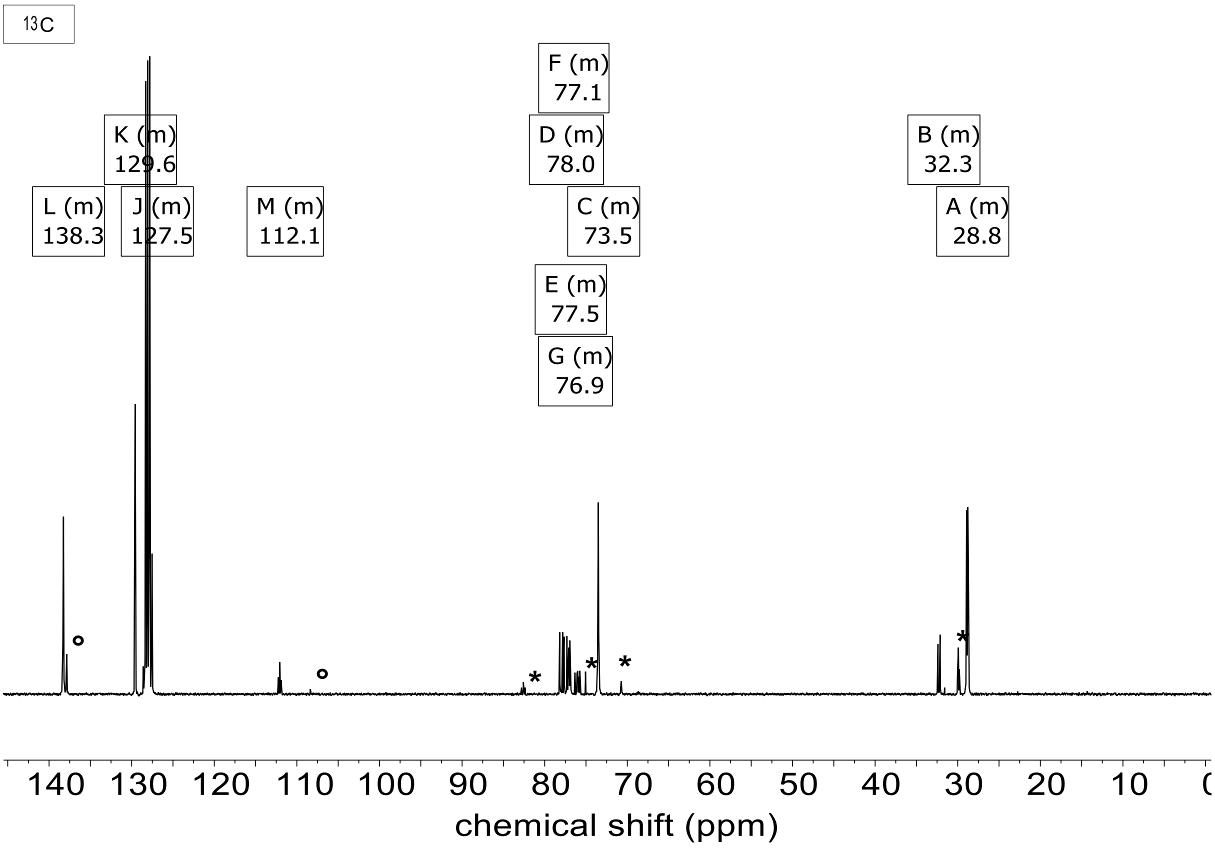

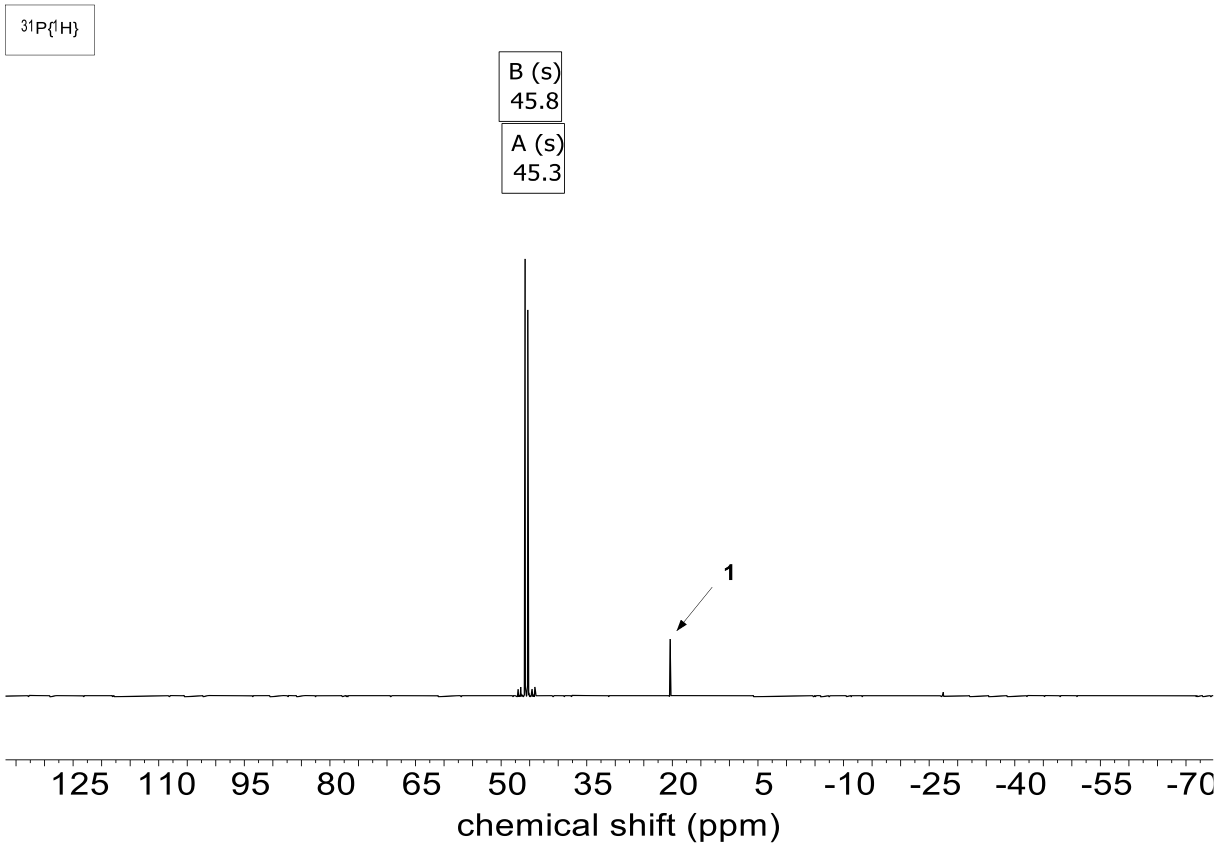
31P{1H}
B (s)
45.8
A (s)
45.3
1
125 110 95 80 65 50 35 20 5 -10 -25 -40 -55 -70
chemical shift (ppm)

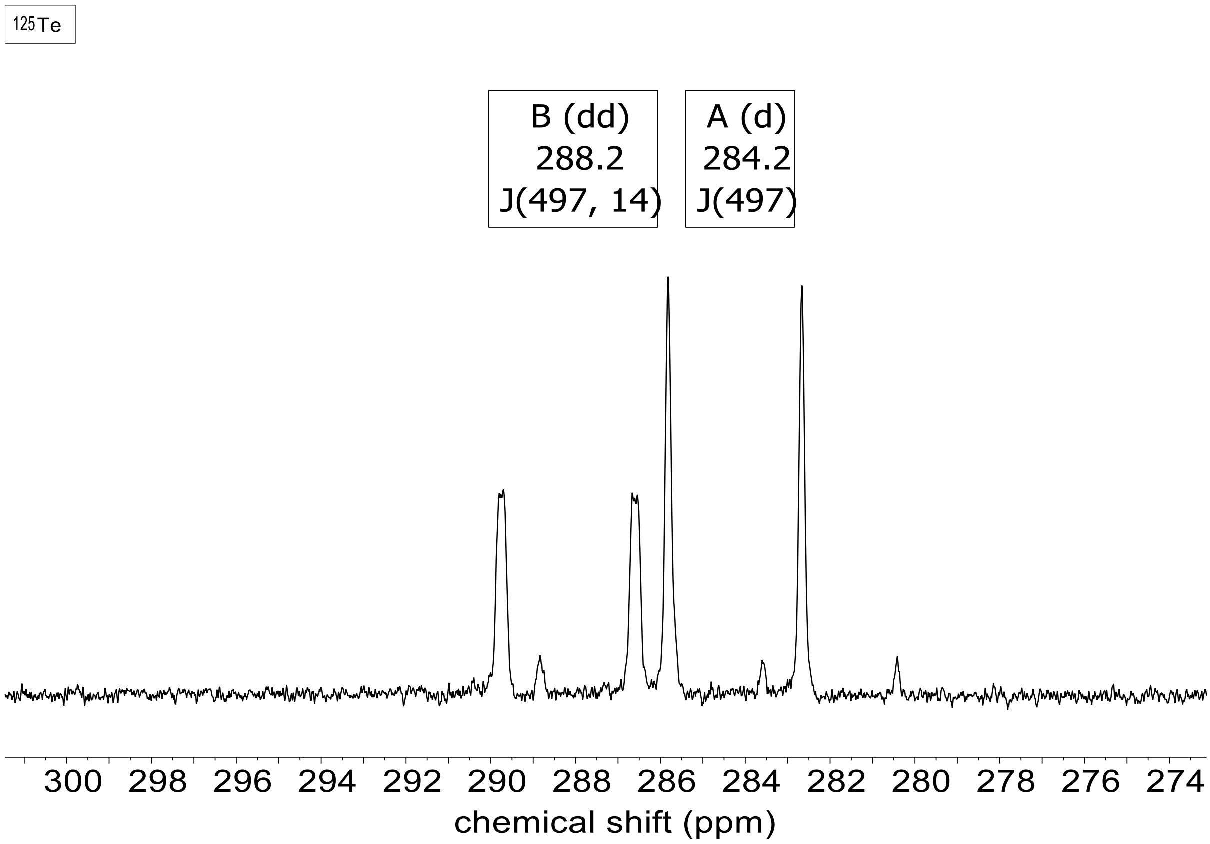
125Te
B (dd)
288.2
J(497, 14)
A (d)
284.2
J(497)
300 298 296 294 292 290 288 286 284 282 280 278 276 274
chemical shift (ppm)

NMR-spectra of **4a**:

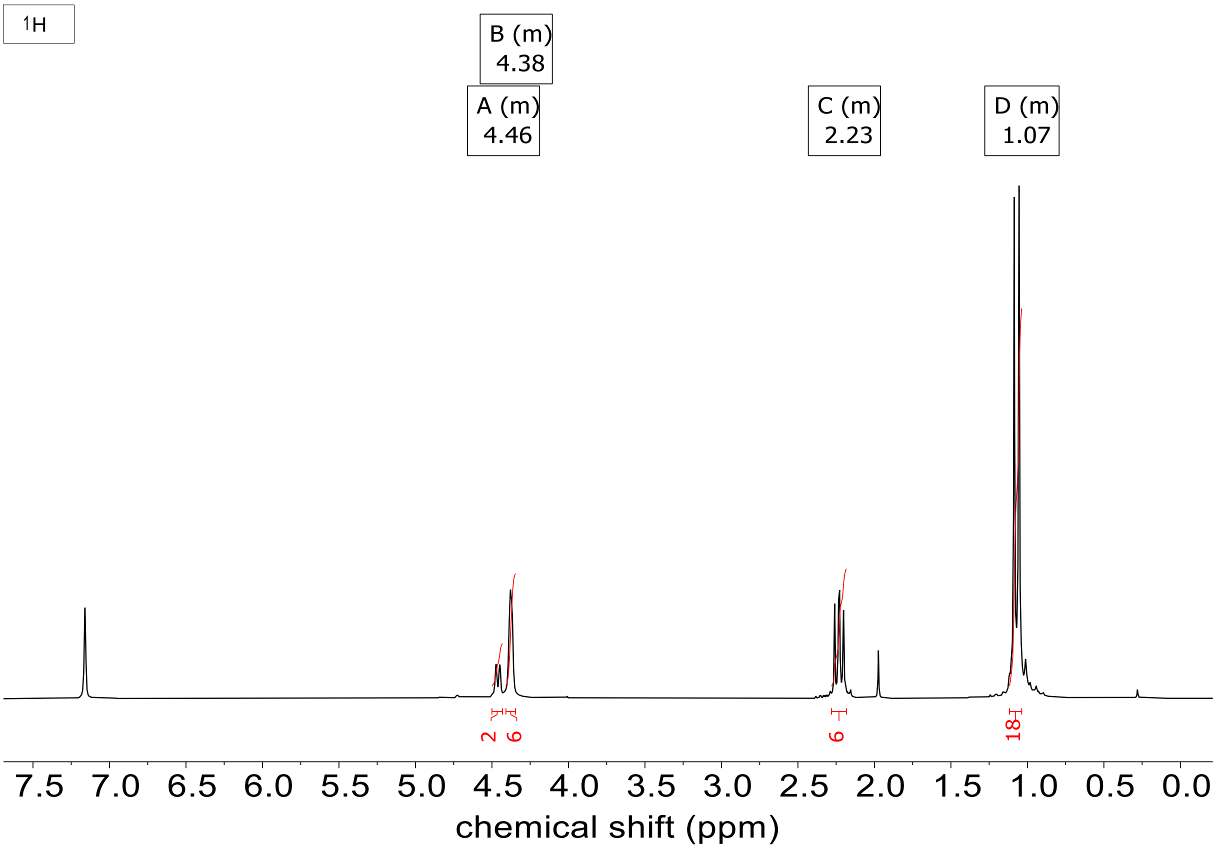

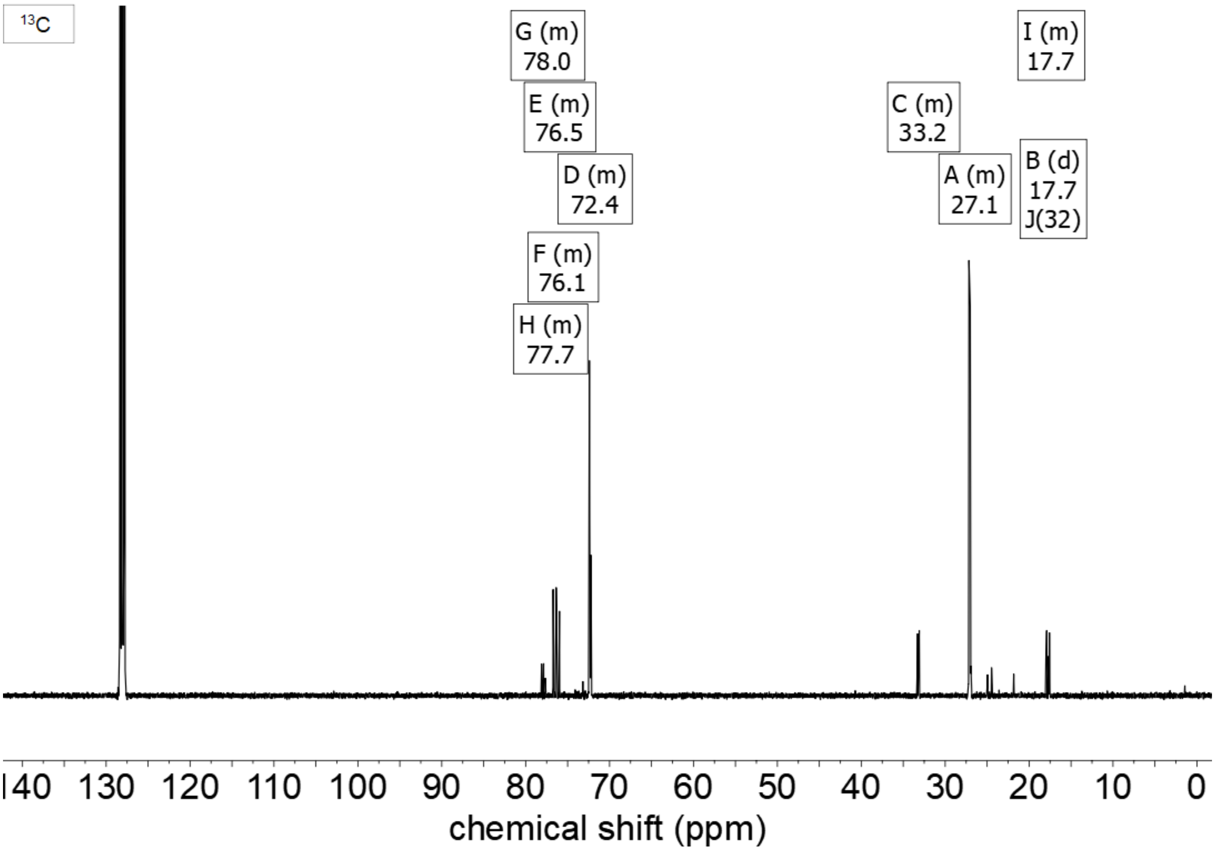

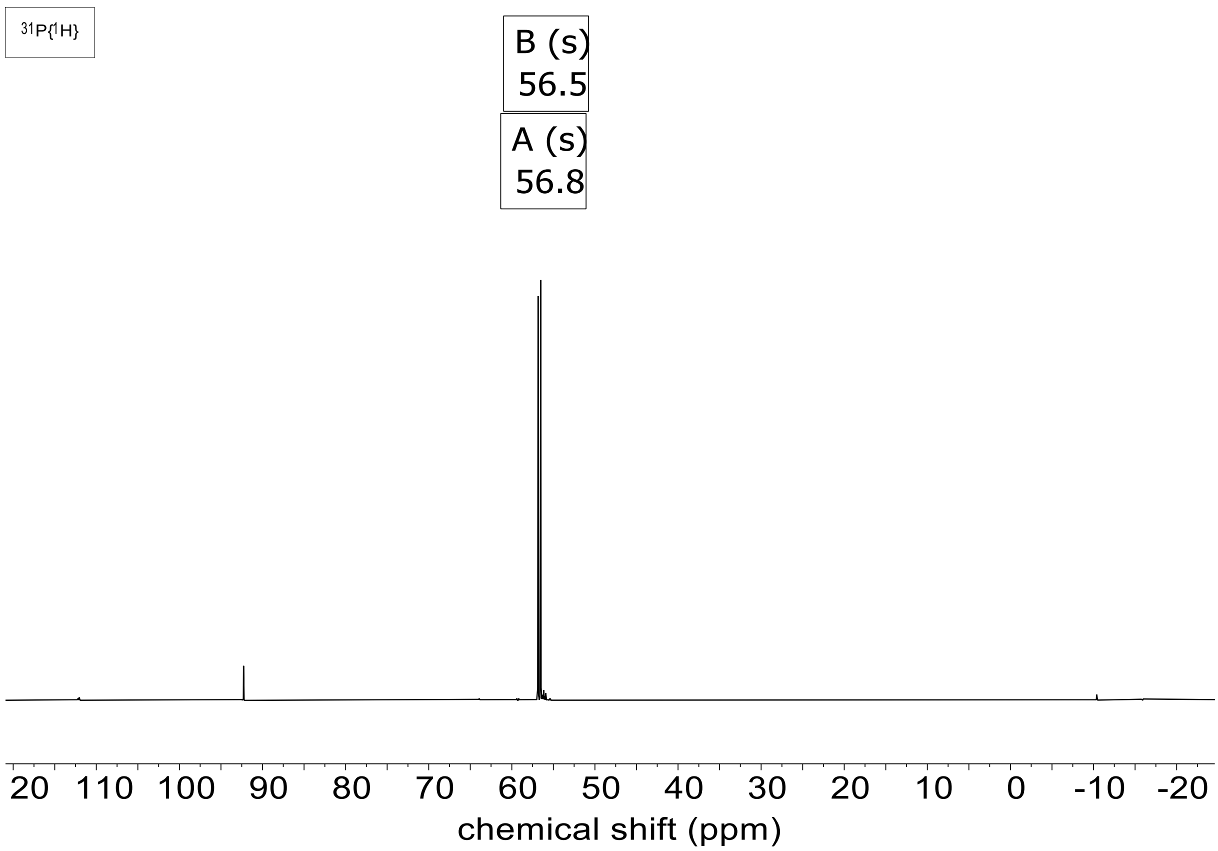

NMR-spectra of **4b**:

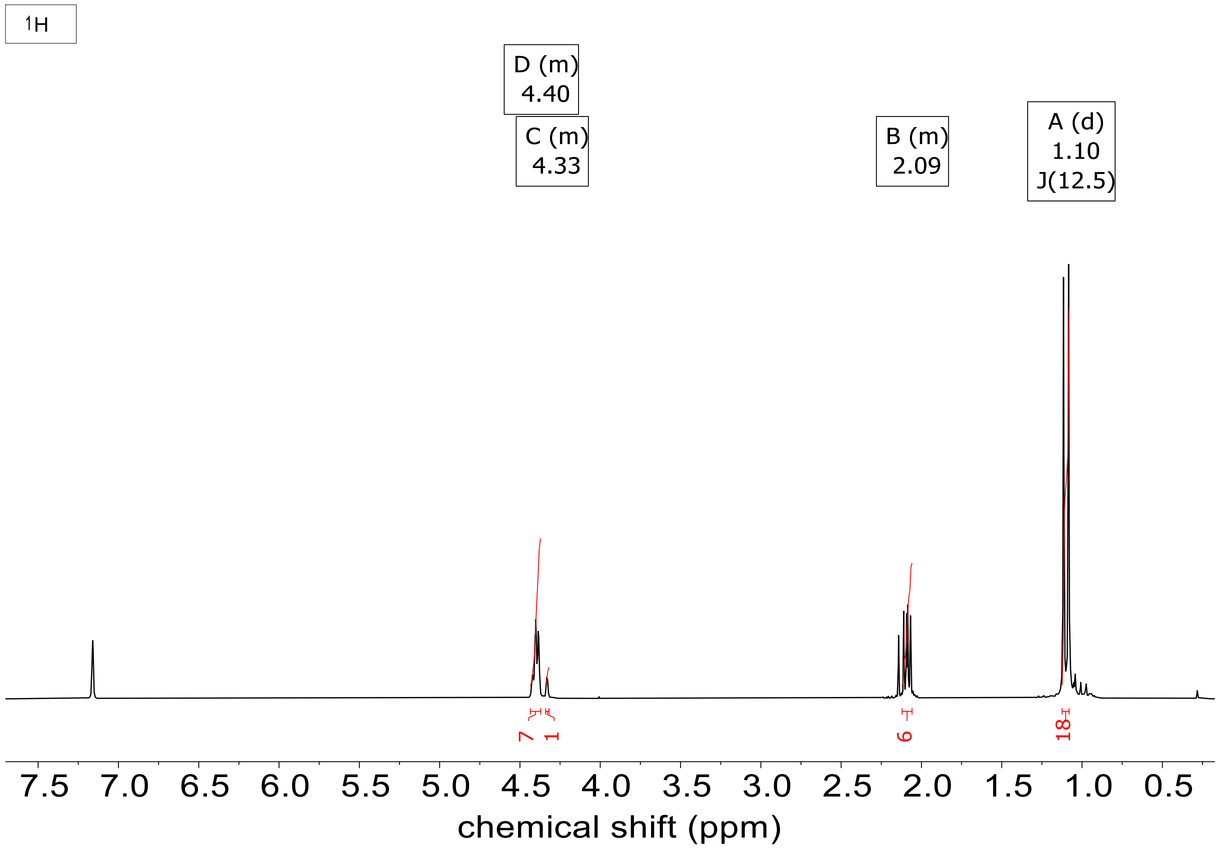

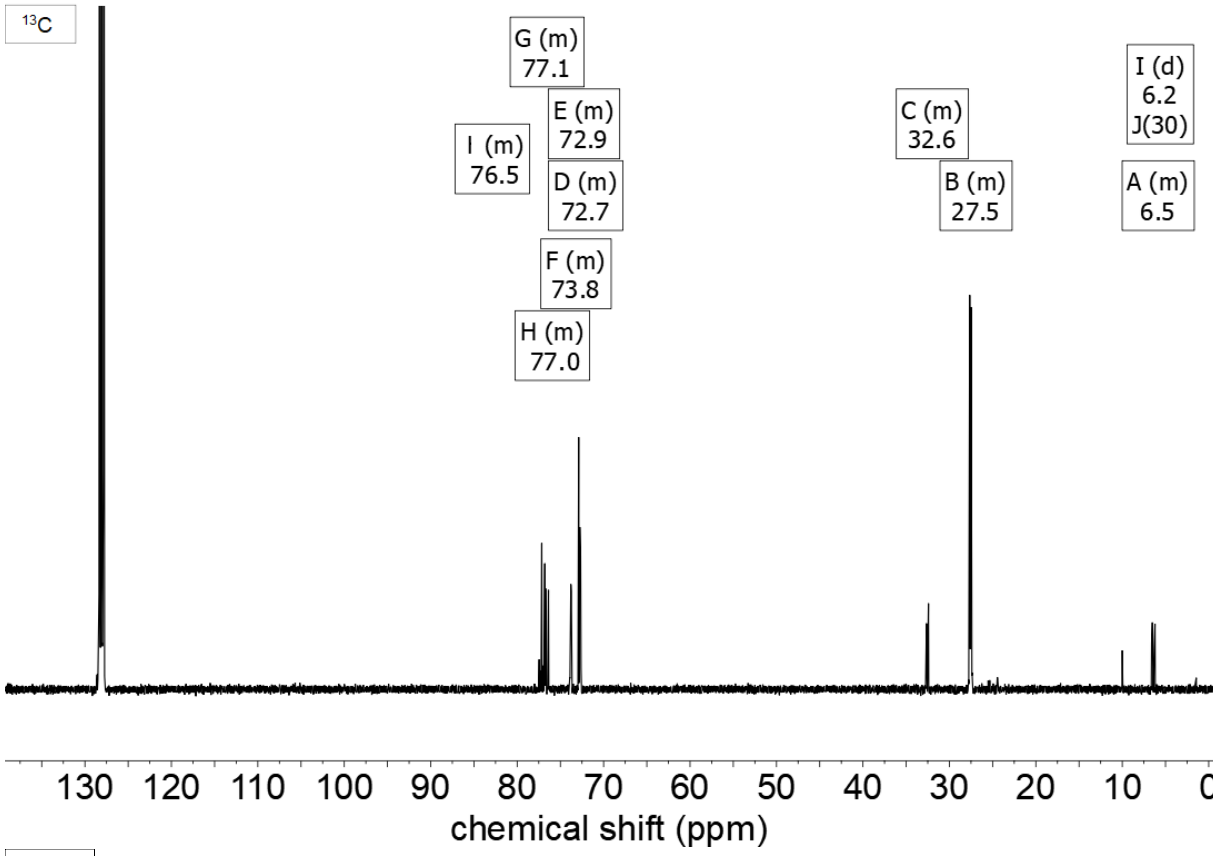
13C
G (m)
77.1
E (m)
72.9
I (m)
76.5
D (m)
72.7
F (m)
73.8
H (m)
77.0
C (m)
32.6
B (m)
27.5
I (d)
6.2
J(30)
A (m)
6.5
130 120 110 100 90 80 70 60 50 40 30 20 10
chemical shift (ppm)

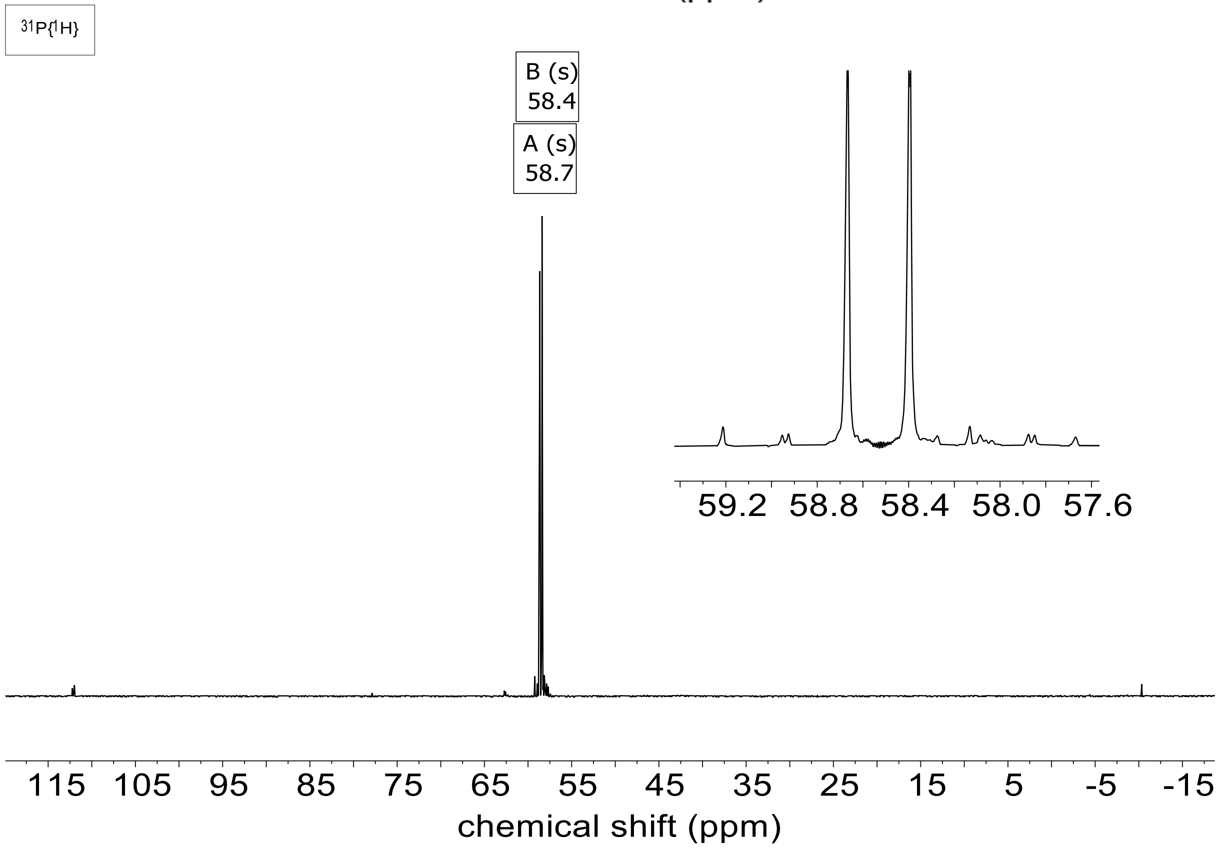
31P{1H}
B (s)
58.4
A (s)
58.7
59.2 58.8 58.4 58.0 57.6
115 105 95 85 75 65 55 45 35 25 15 5 -5 -15
chemical shift (ppm)

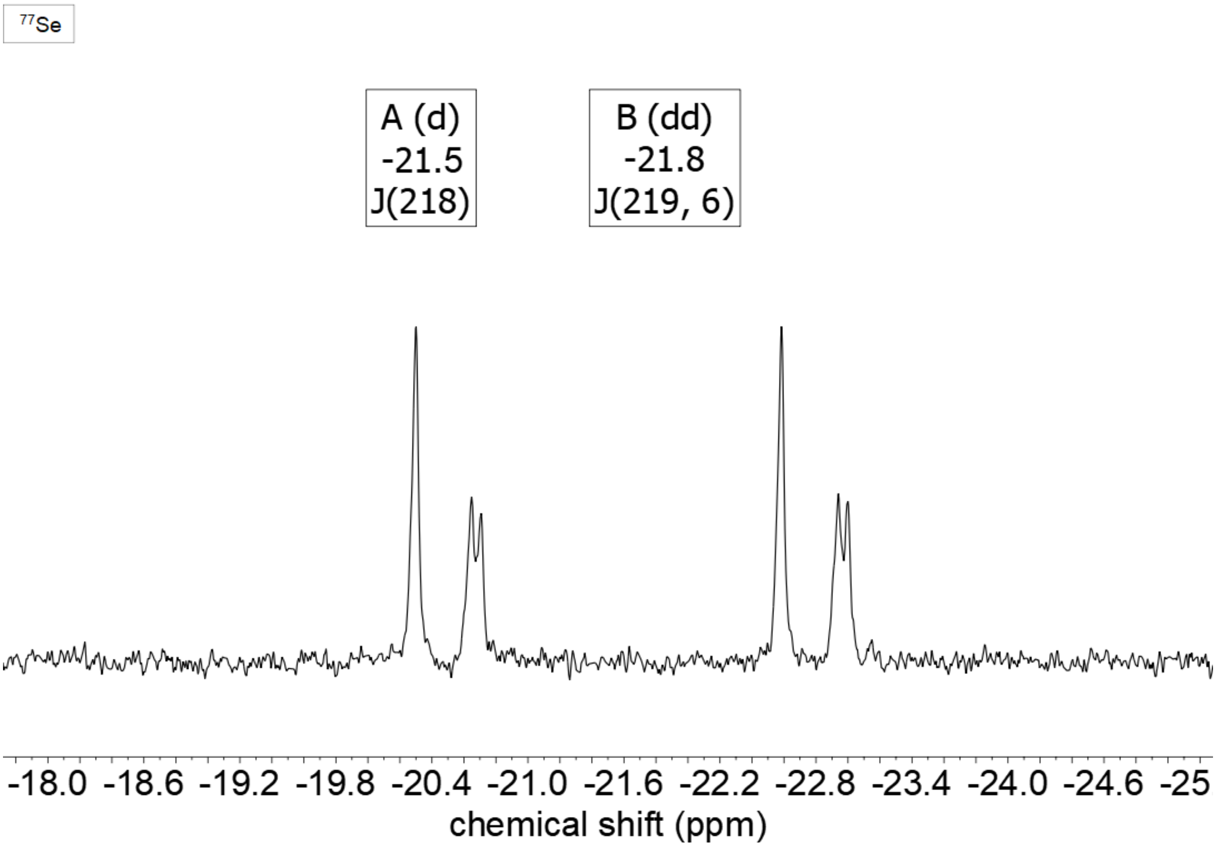

NMR-spectra of **5**:

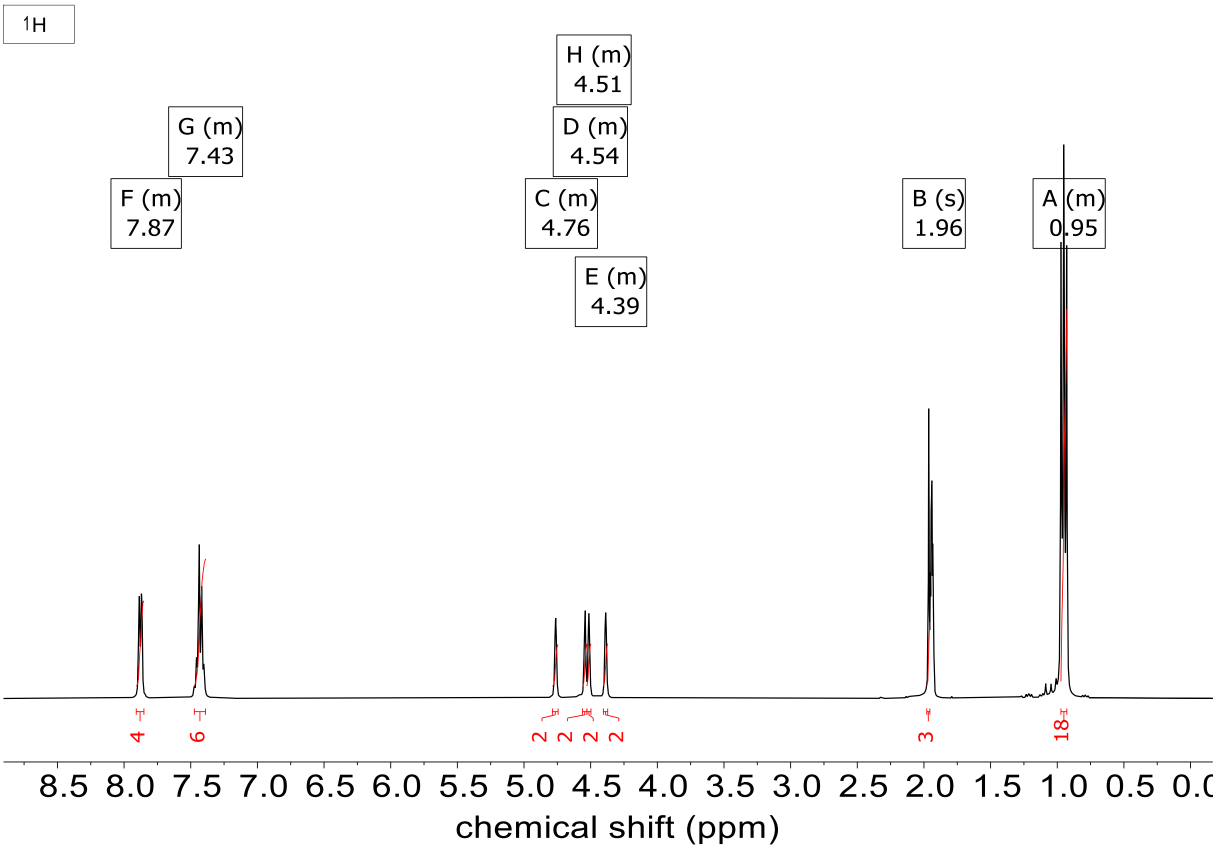

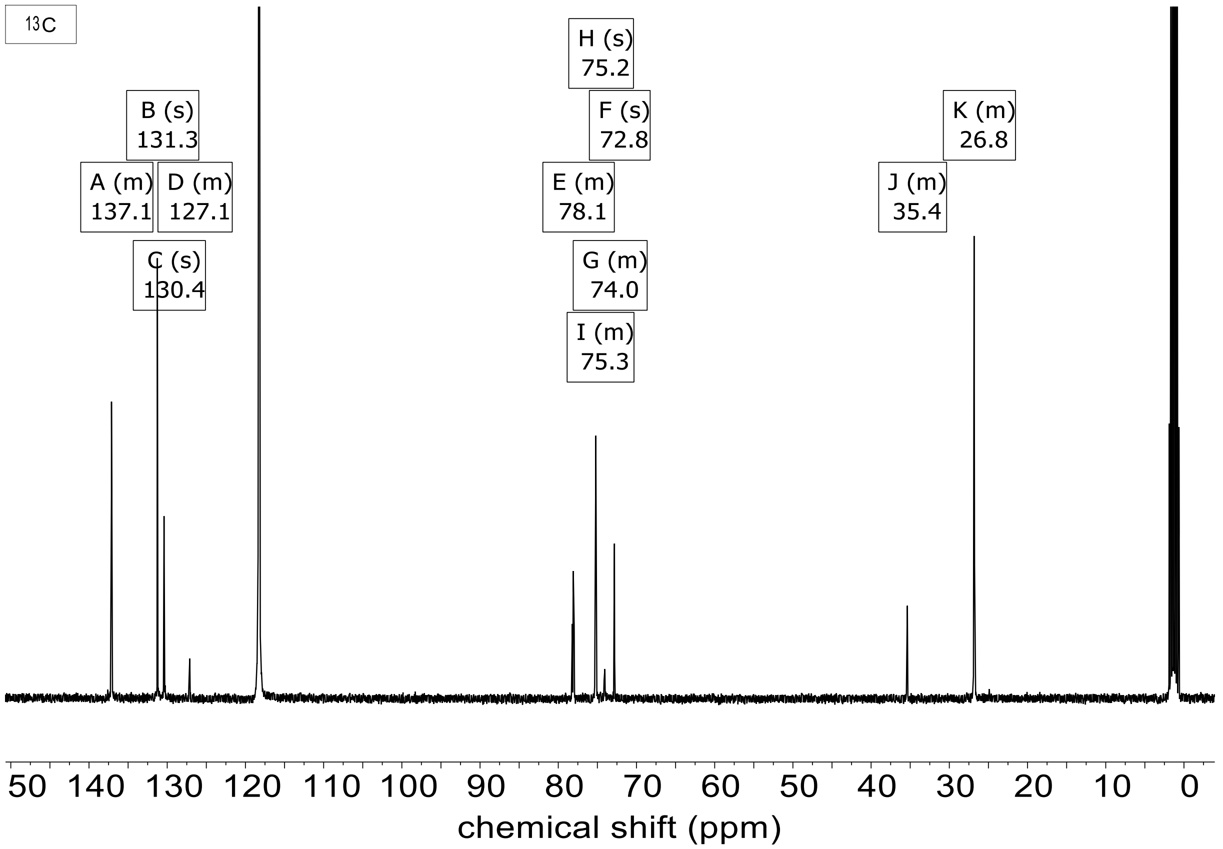
13C
A (m) 137.1
B (s) 131.3
C (s) 130.4
D (m) 127.1
E (m) 78.1
H (s) 75.2
I (m) 75.3
G (m) 74.0
F (s) 72.8
J (m) 35.4
K (m) 26.8
50 140 130 120 110 100 90 80 70 60 50 40 30 20 10 0
chemical shift (ppm)

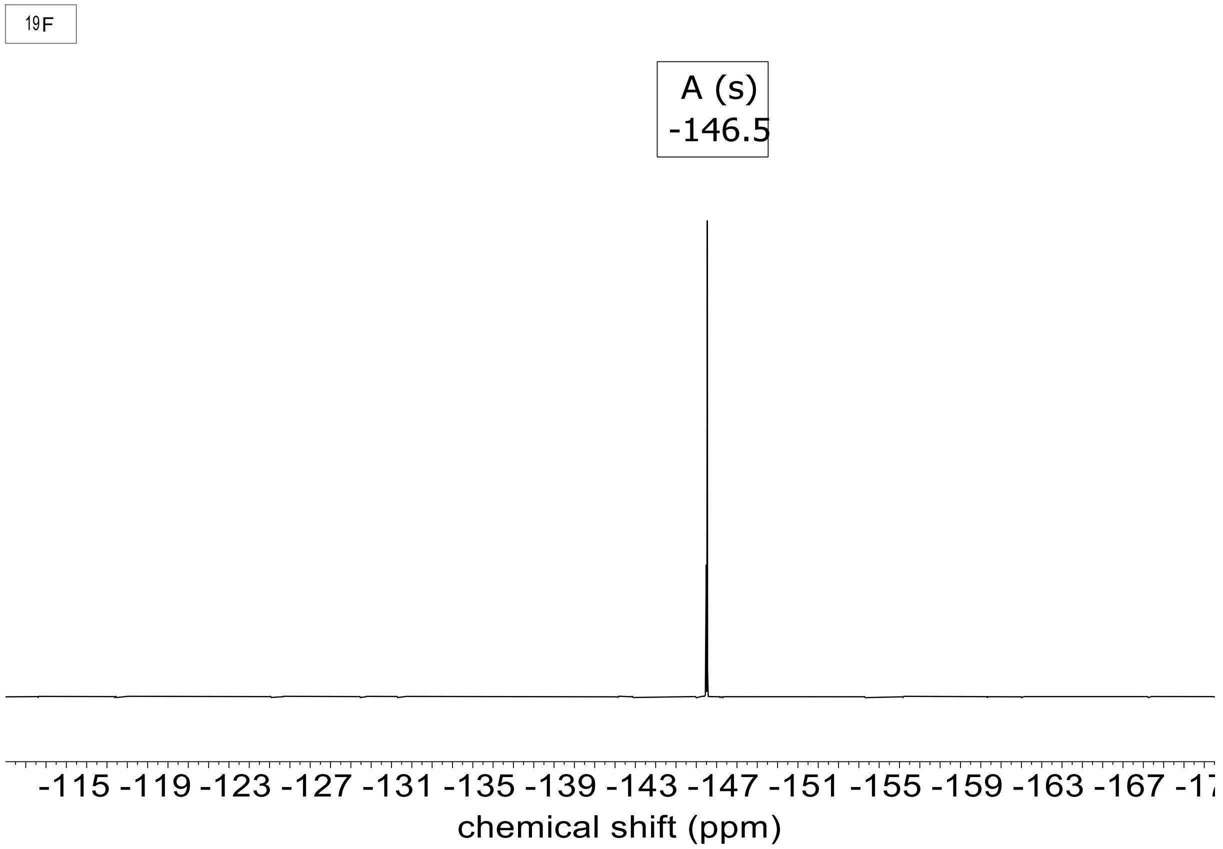
19F
A (s) -146.5
-115 -119 -123 -127 -131 -135 -139 -143 -147 -151 -155 -159 -163 -167
chemical shift (ppm)

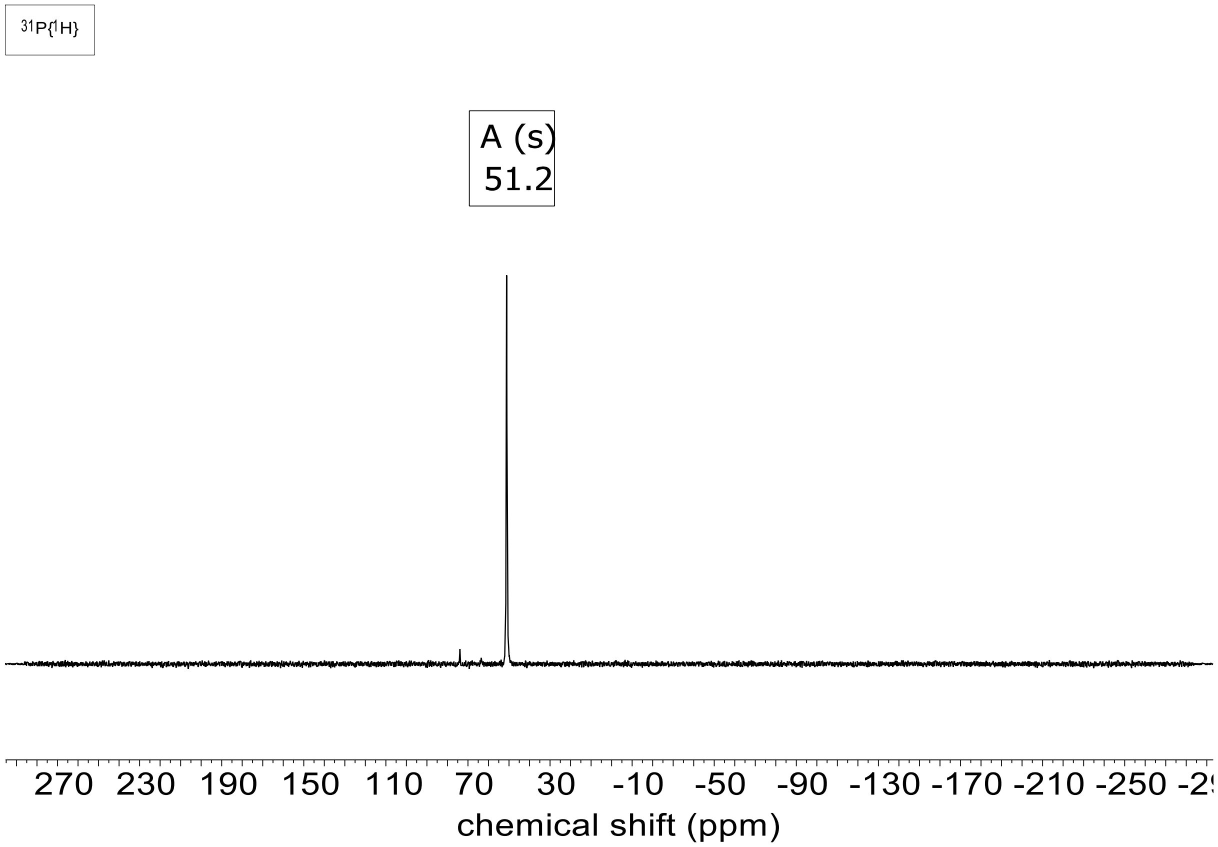
31P{1H}
A (s)
51.2
270 230 190 150 110 70 30 -10 -50 -90 -130 -170 -210 -250
chemical shift (ppm)

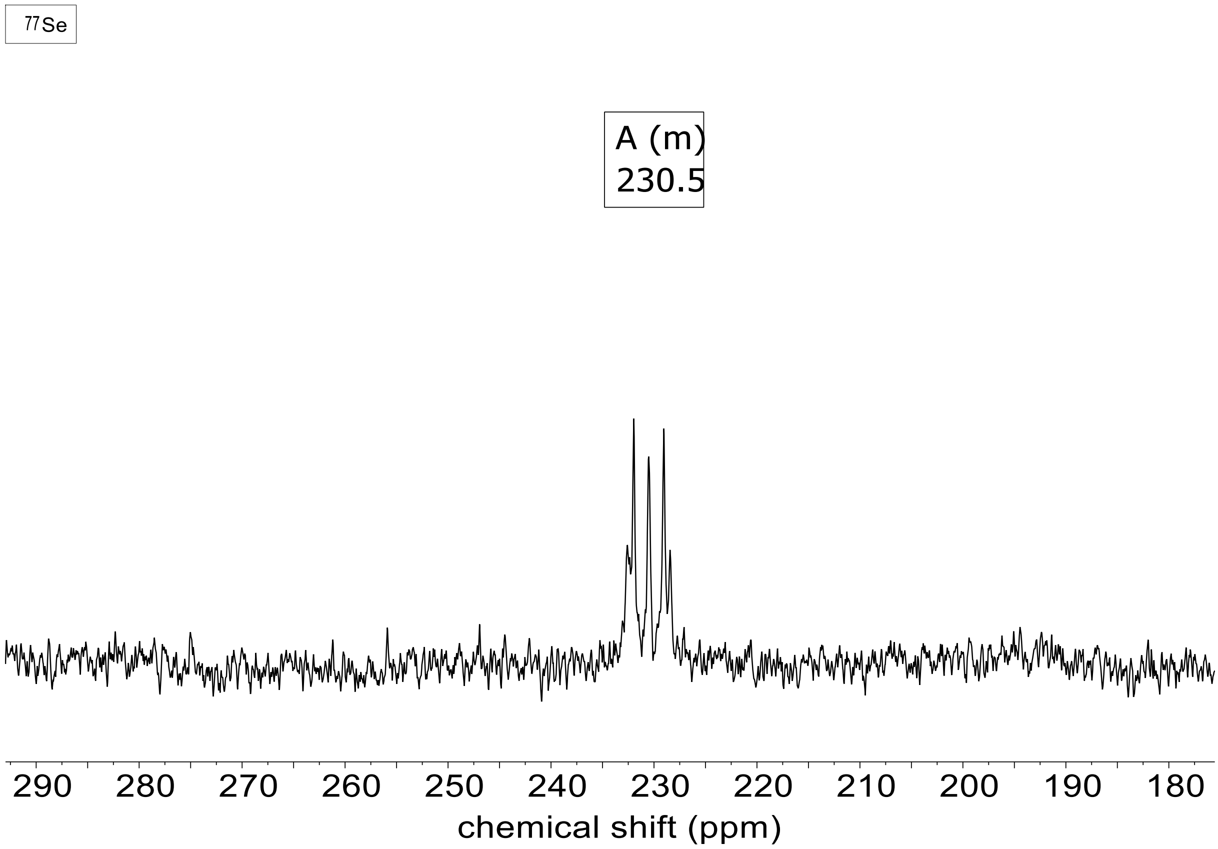
77Se
A (m)
230.5
290 280 270 260 250 240 230 220 210 200 190 180
chemical shift (ppm)

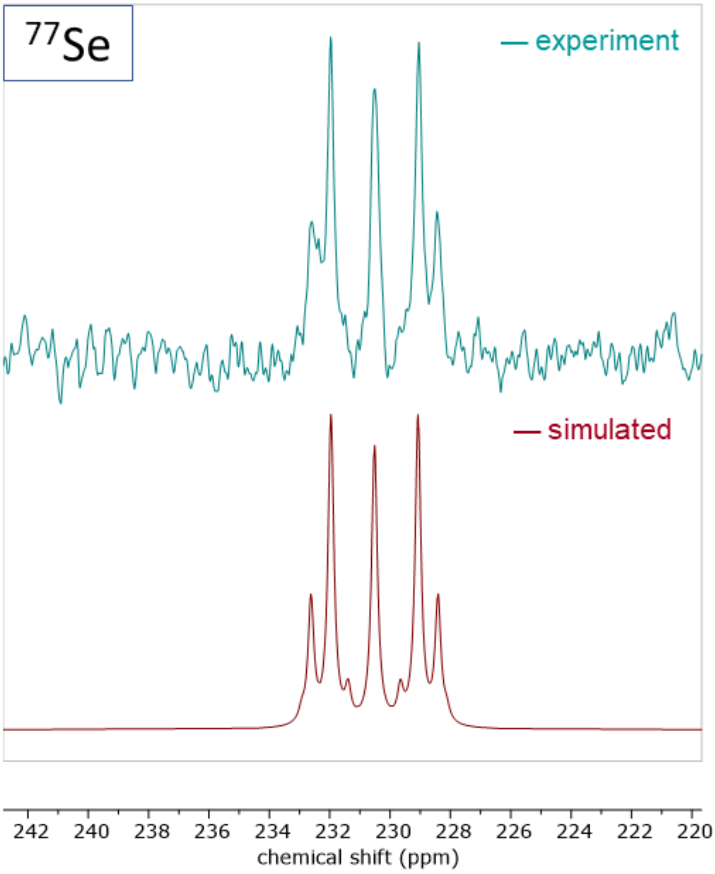

Figure S2: Measured (top) and simulated (bottom) ^{77}Se-NMR spectrum of silver(I) complex 6 (MeCN-*d*3, 300 K). Following coupling constants were used for the simulation: $^{1}J_{PSe}$ = 276 Hz, $^{2}J_{PP}$ = 145 Hz, $^{1}J_{P\text{-}(63)Cu}$ = 100 Hz, $^{1}J_{P\text{-}(65)Cu}$ = 150 Hz, $^{3}J_{PSe}$ = 0 Hz, $^{2}J_{Se\text{-}(63)Cu}$ = $^{2}J_{Se\text{-}(65)Cu}$ = 0 Hz.

NMR-spectra of **6**:

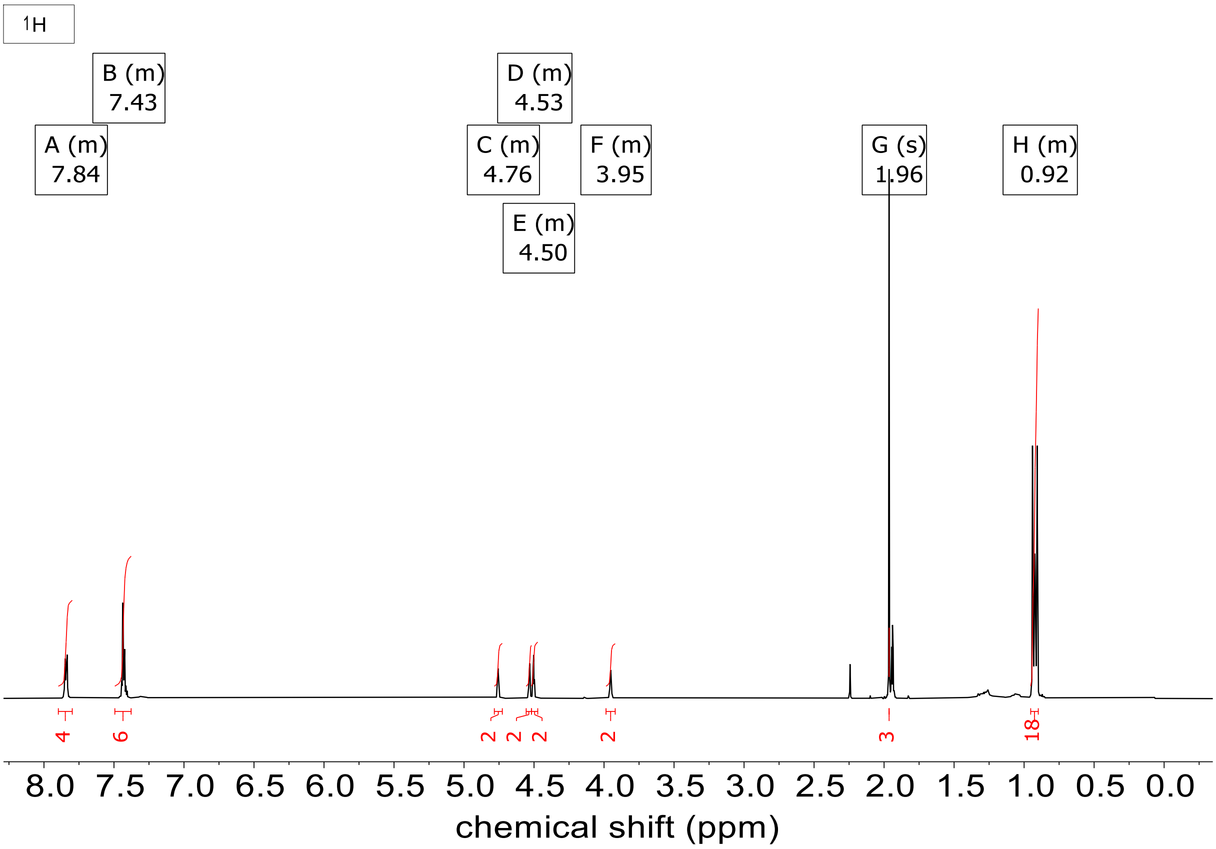

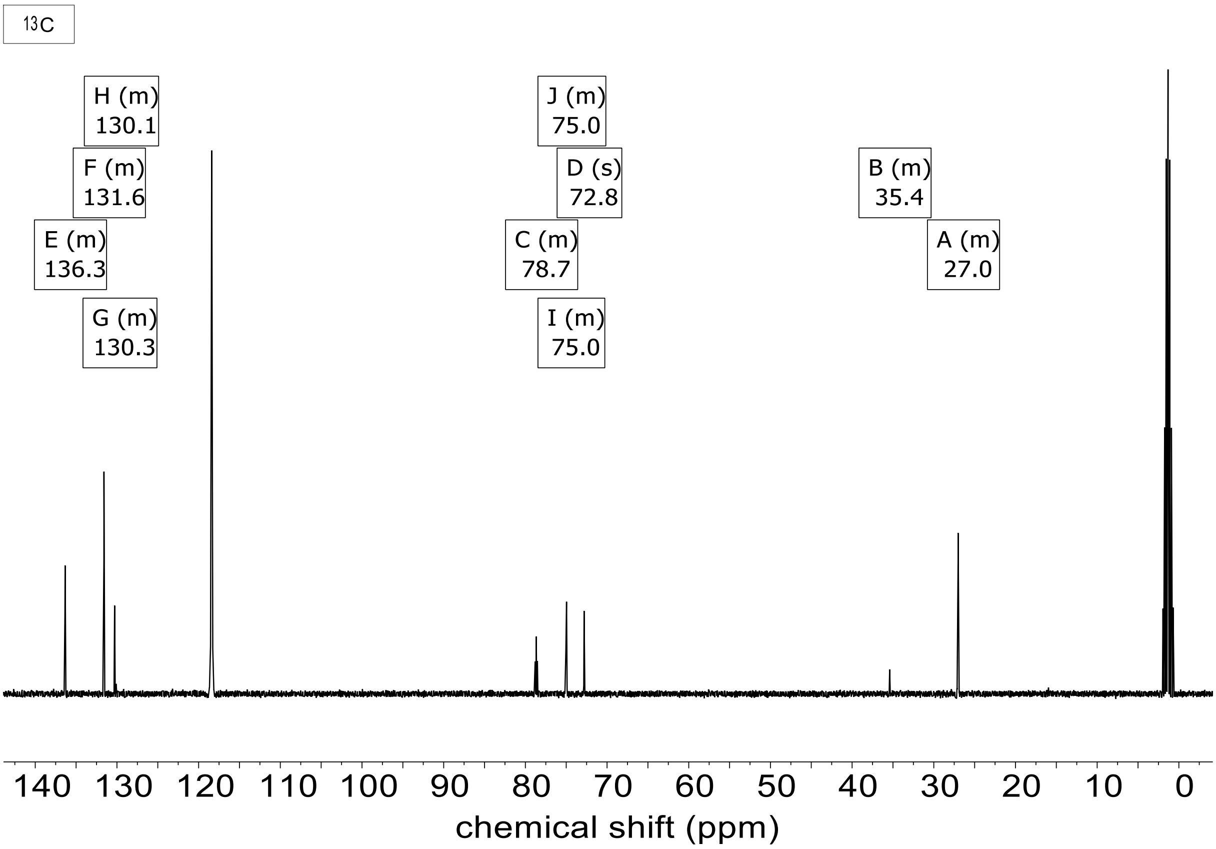

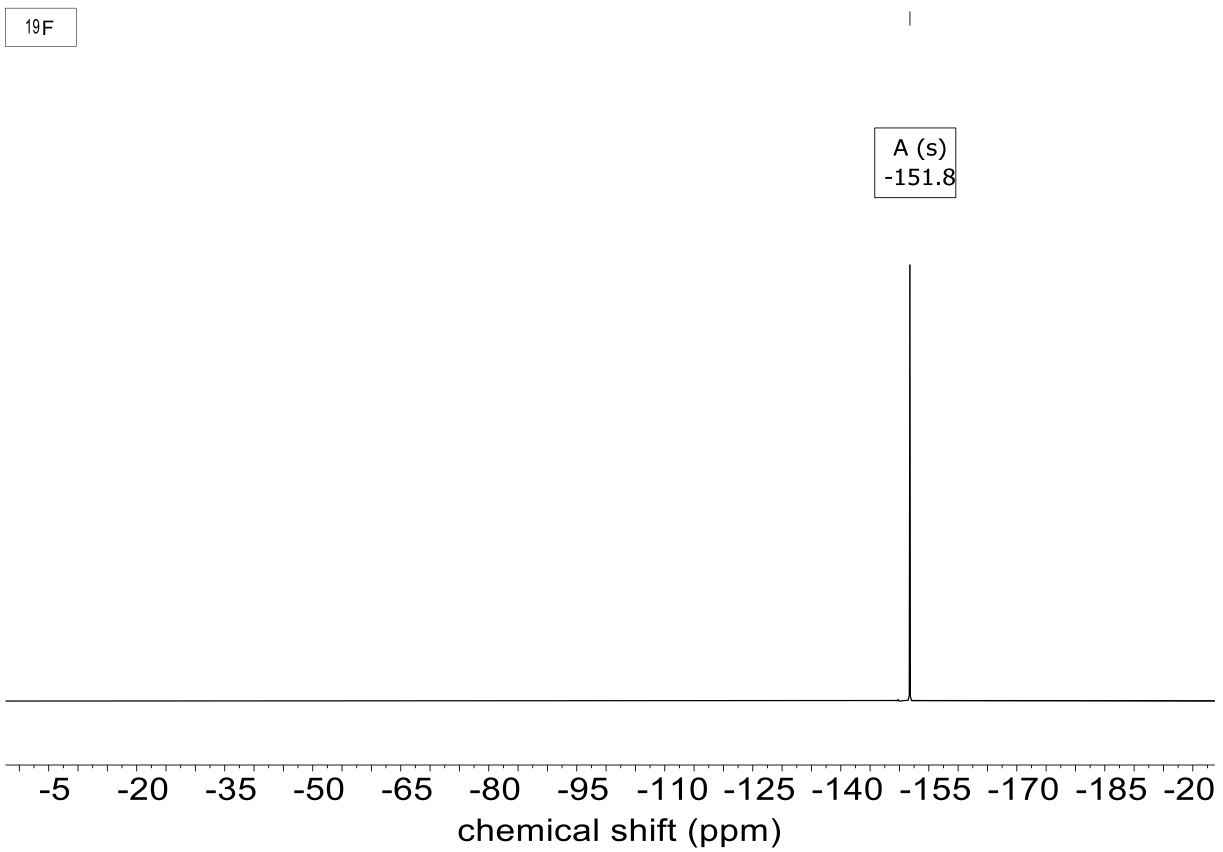

19F
A (s)
-151.8
-5 -20 -35 -50 -65 -80 -95 -110 -125 -140 -155 -170 -185 -20
chemical shift (ppm)

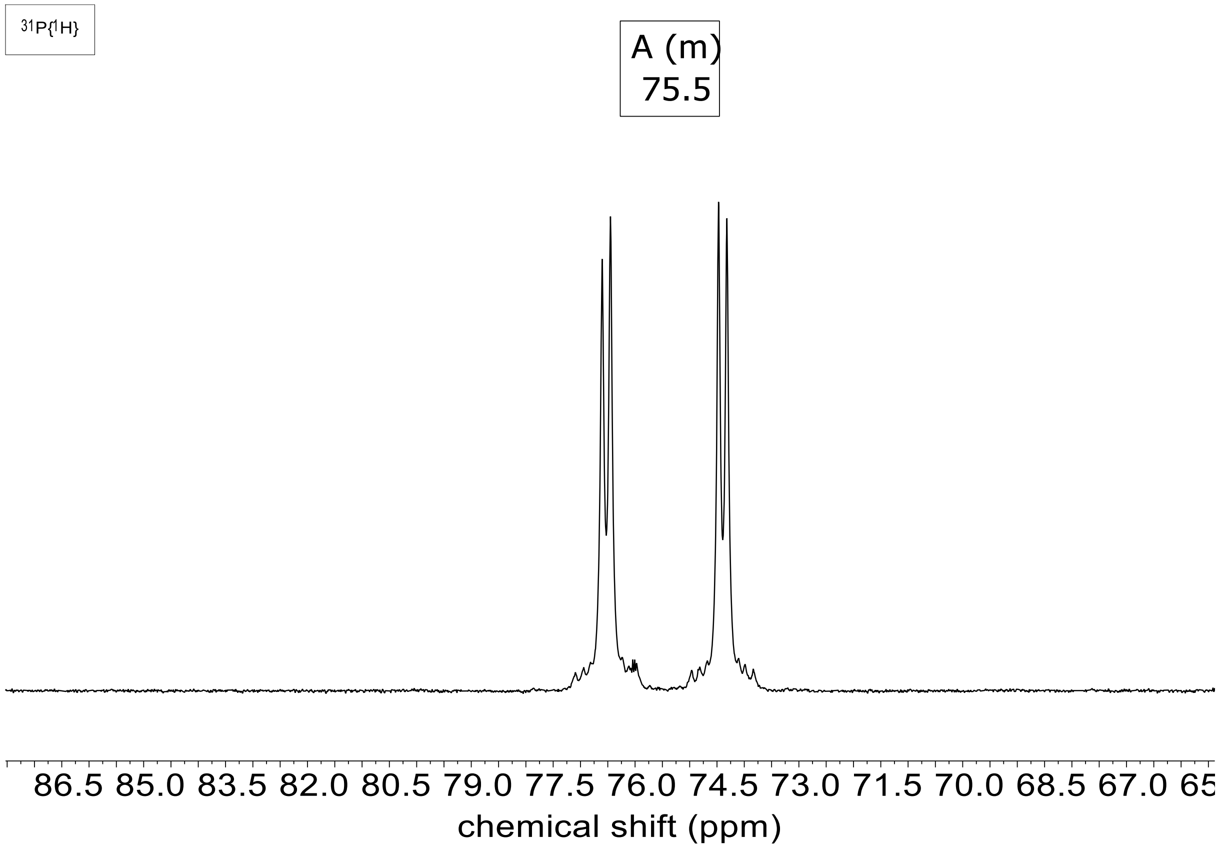

31P{1H}
A (m)
75.5
86.5 85.0 83.5 82.0 80.5 79.0 77.5 76.0 74.5 73.0 71.5 70.0 68.5 67.0 65
chemical shift (ppm)

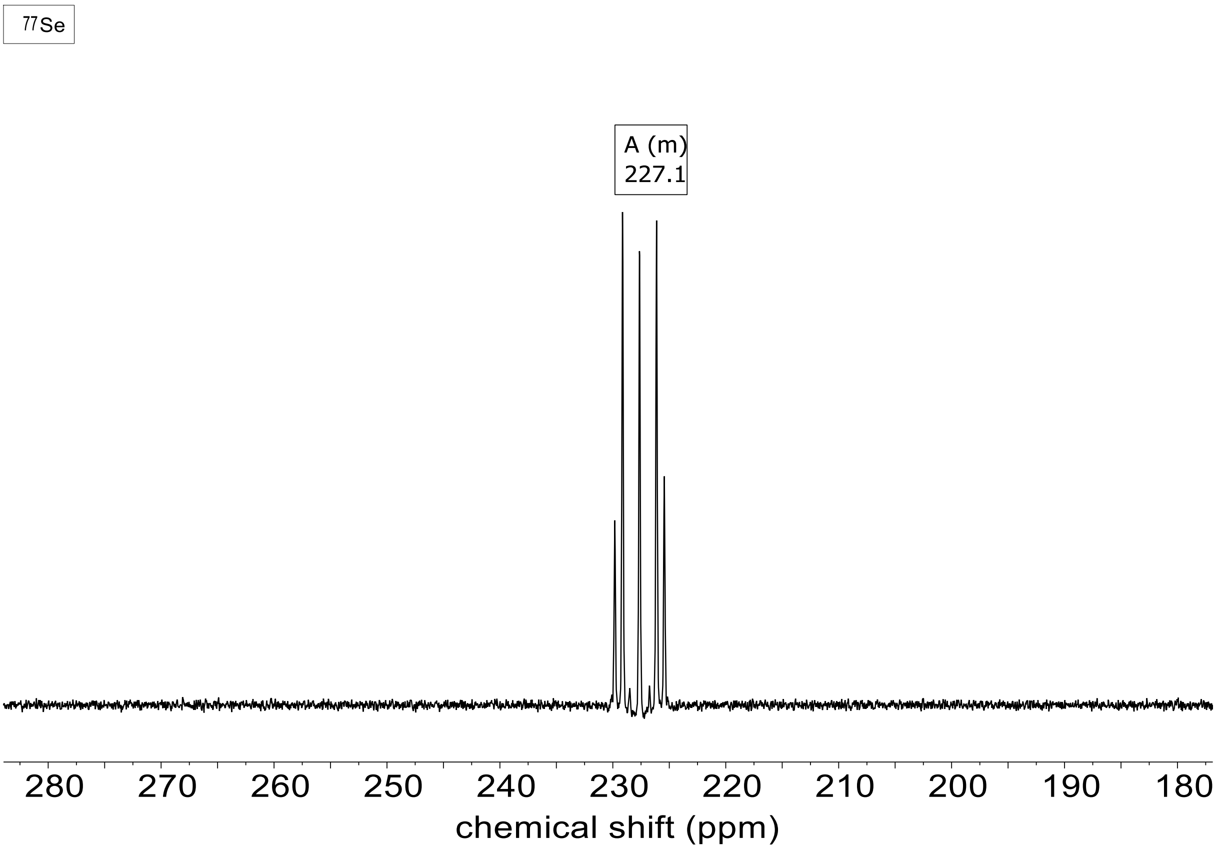

NMR-spectra of **7**:

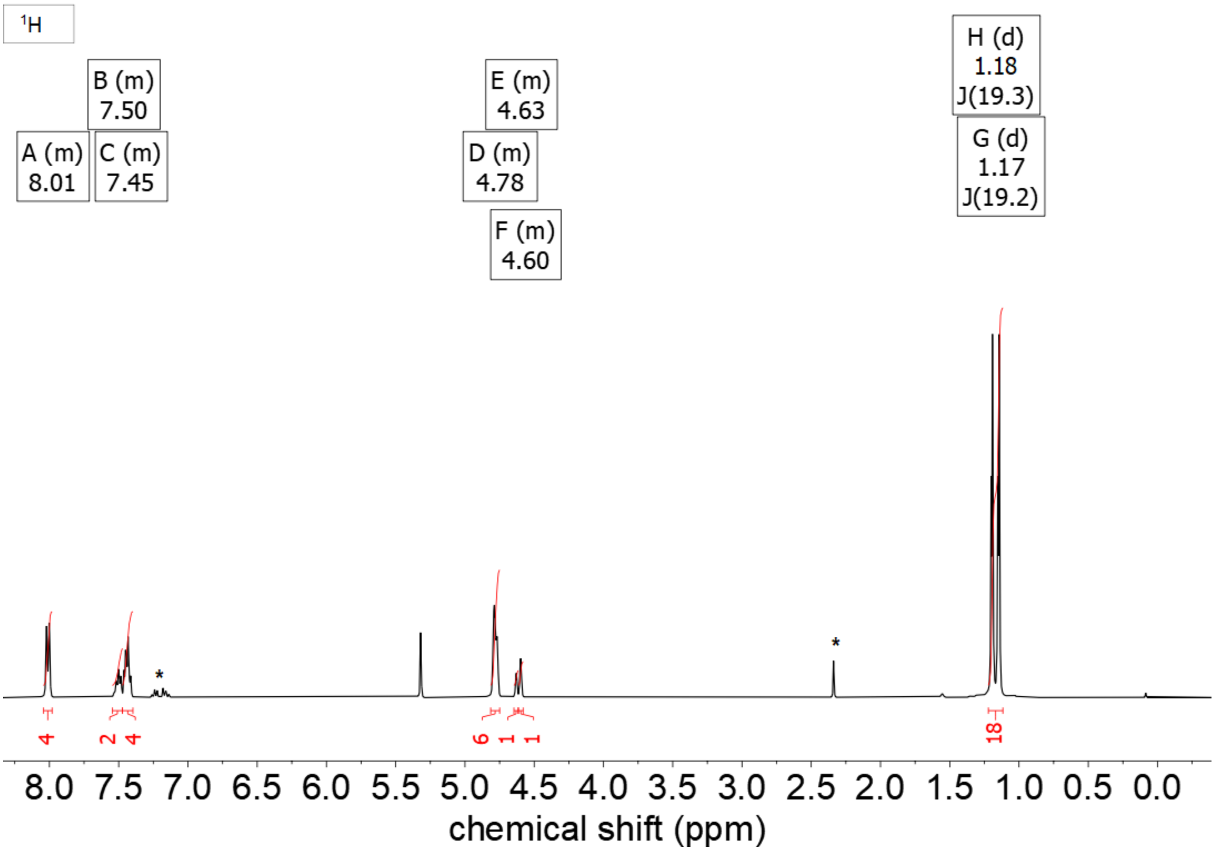

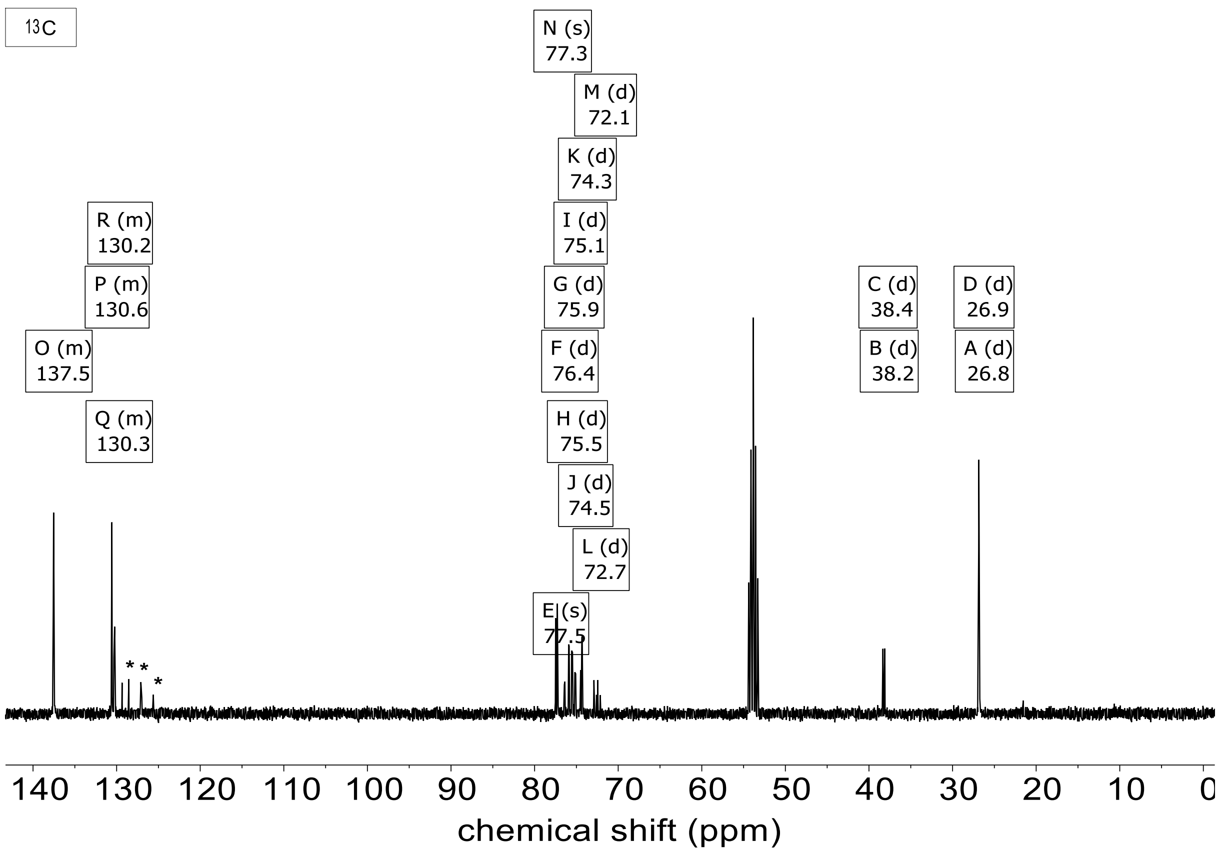
13C
N (s) 77.3
M (d) 72.1
K (d) 74.3
I (d) 75.1
R (m) 130.2
P (m) 130.6
O (m) 137.5
Q (m) 130.3
G (d) 75.9
F (d) 76.4
H (d) 75.5
J (d) 74.5
L (d) 72.7
E (s) 77.5
C (d) 38.4
B (d) 38.2
D (d) 26.9
A (d) 26.8
* * *
140 130 120 110 100 90 80 70 60 50 40 30 20 10 0
chemical shift (ppm)

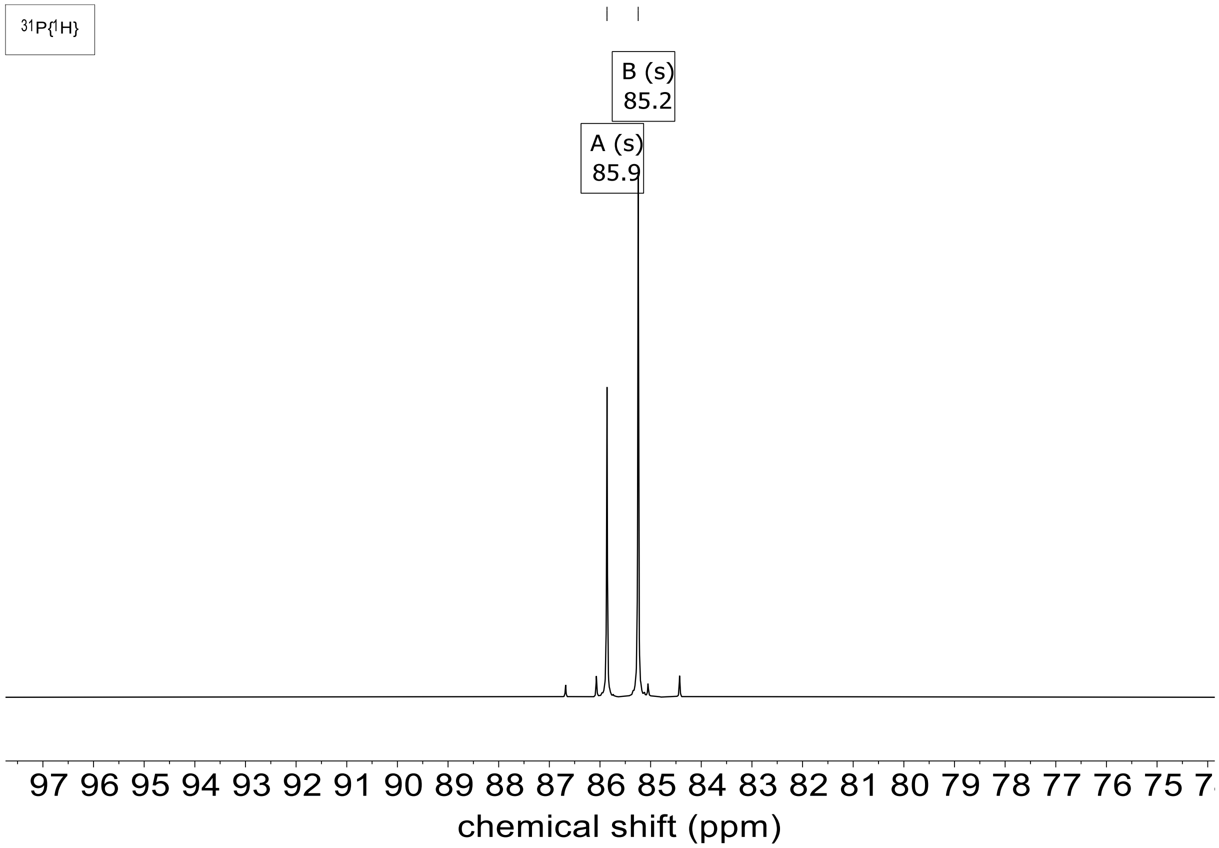
31P{1H}
B (s) 85.2
A (s) 85.9
97 96 95 94 93 92 91 90 89 88 87 86 85 84 83 82 81 80 79 78 77 76 75
chemical shift (ppm)

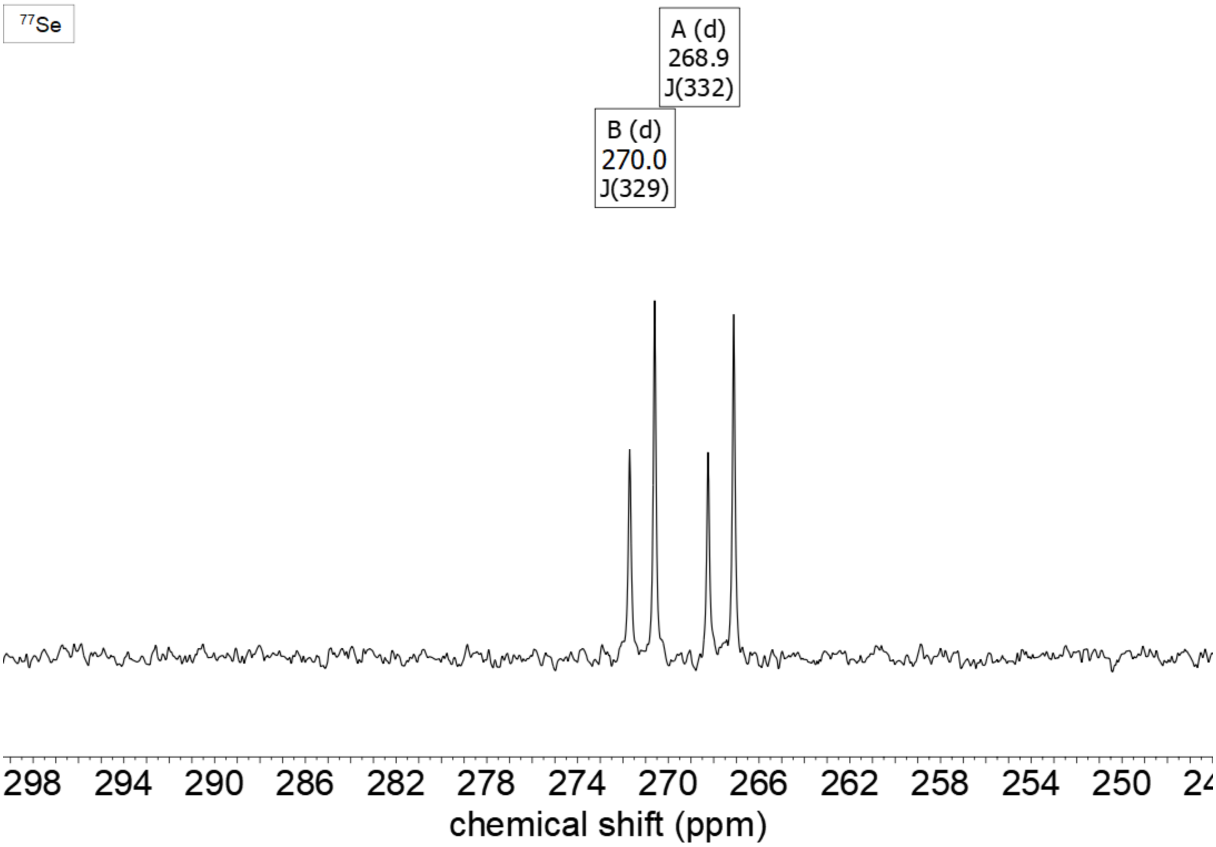

NMR-spectra of **8**:

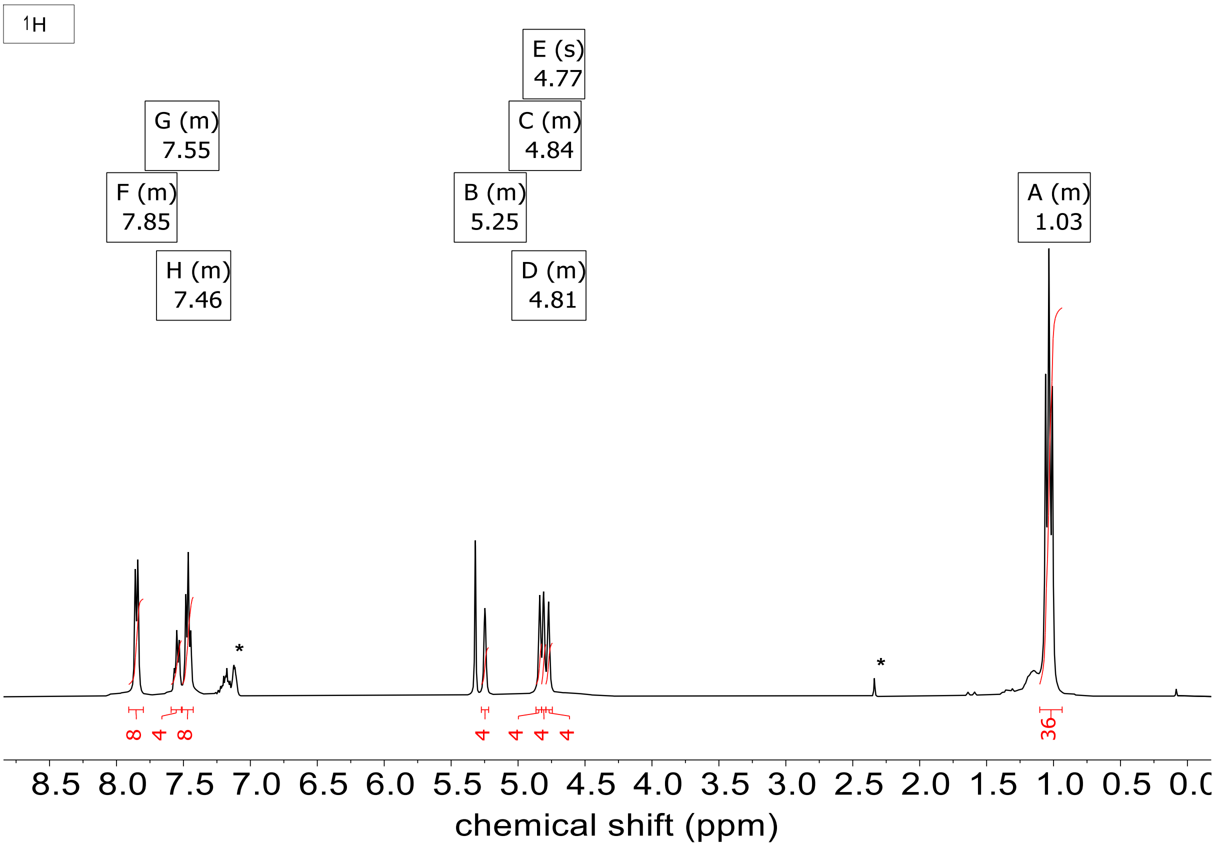

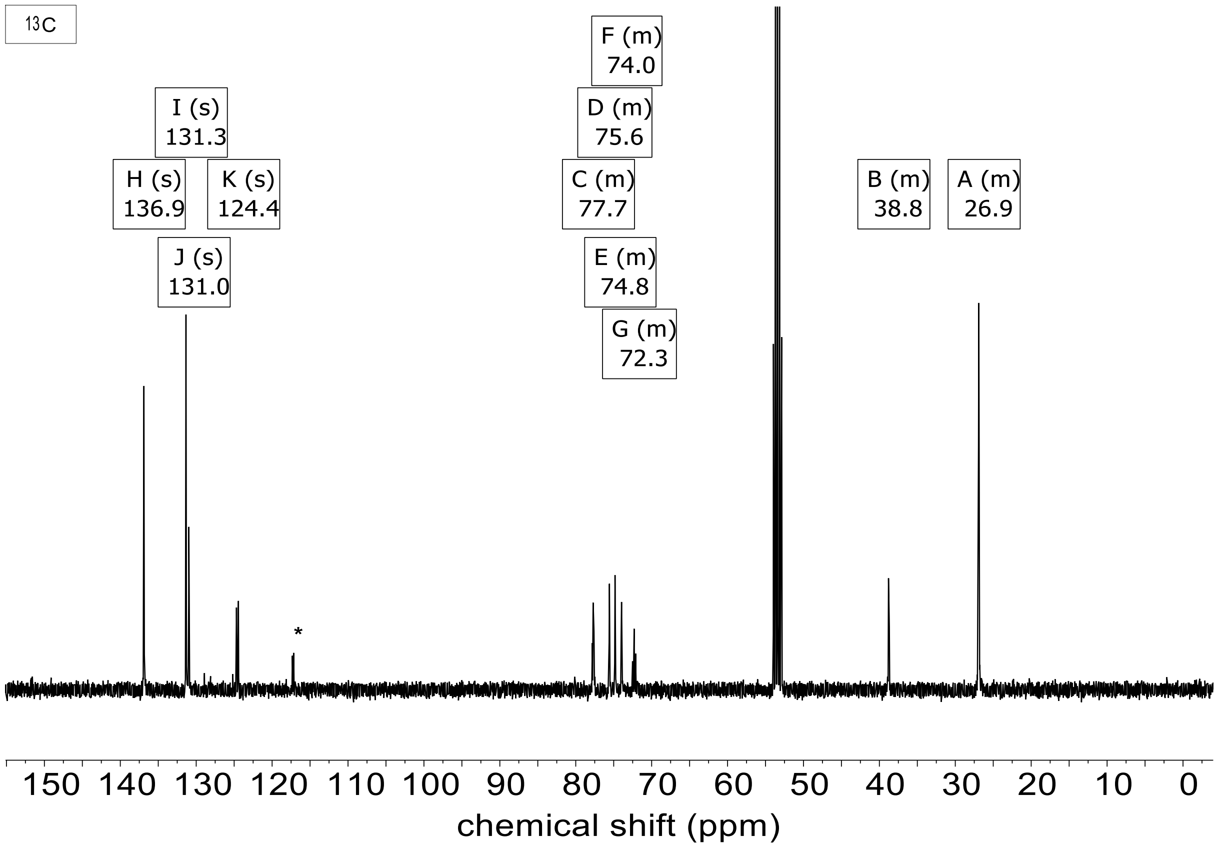
13C
I (s)
131.3
H (s)
136.9
K (s)
124.4
J (s)
131.0
F (m)
74.0
D (m)
75.6
C (m)
77.7
E (m)
74.8
G (m)
72.3
B (m)
38.8
A (m)
26.9
*
150 140 130 120 110 100 90 80 70 60 50 40 30 20 10 0
chemical shift (ppm)

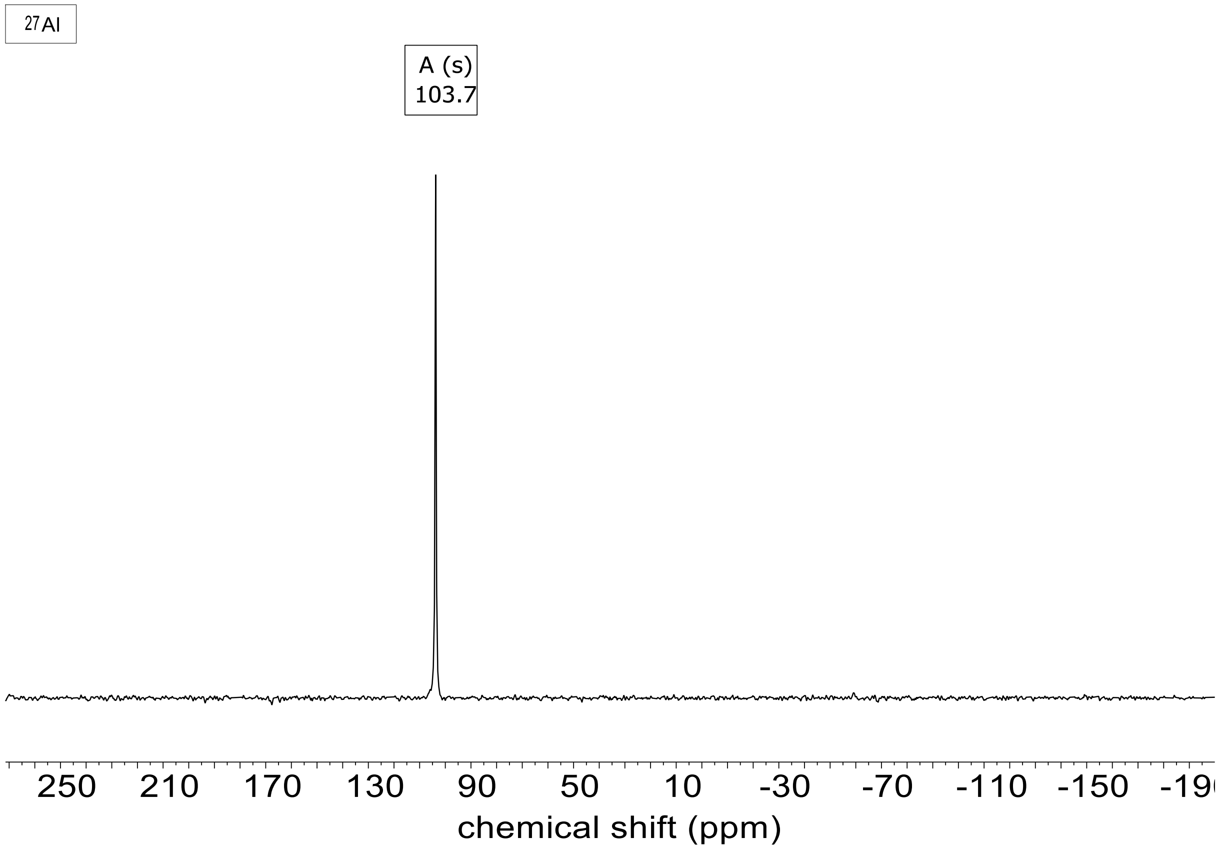
27Al
A (s)
103.7
250 210 170 130 90 50 10 -30 -70 -110 -150
chemical shift (ppm)

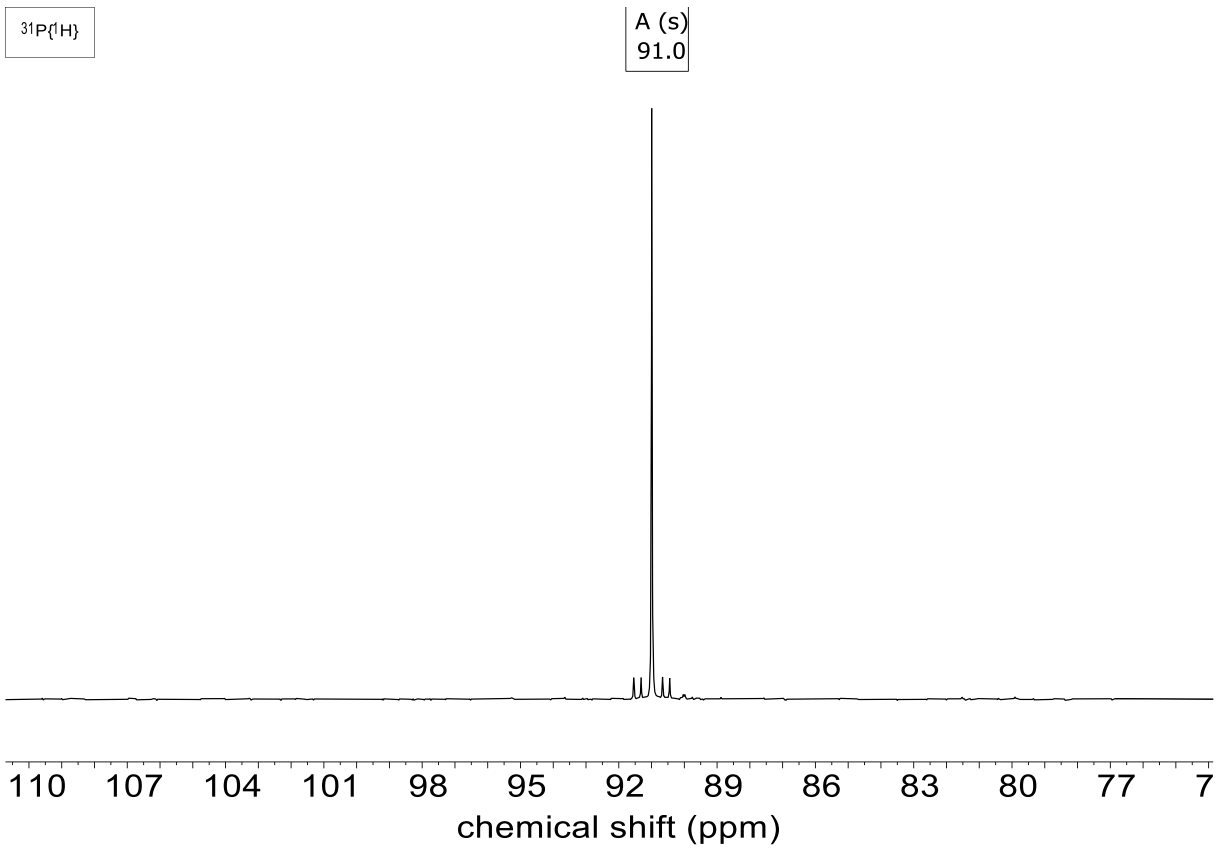
31P{1H}
A (s)
91.0
110 107 104 101 98 95 92 89 86 83 80 77 7
chemical shift (ppm)

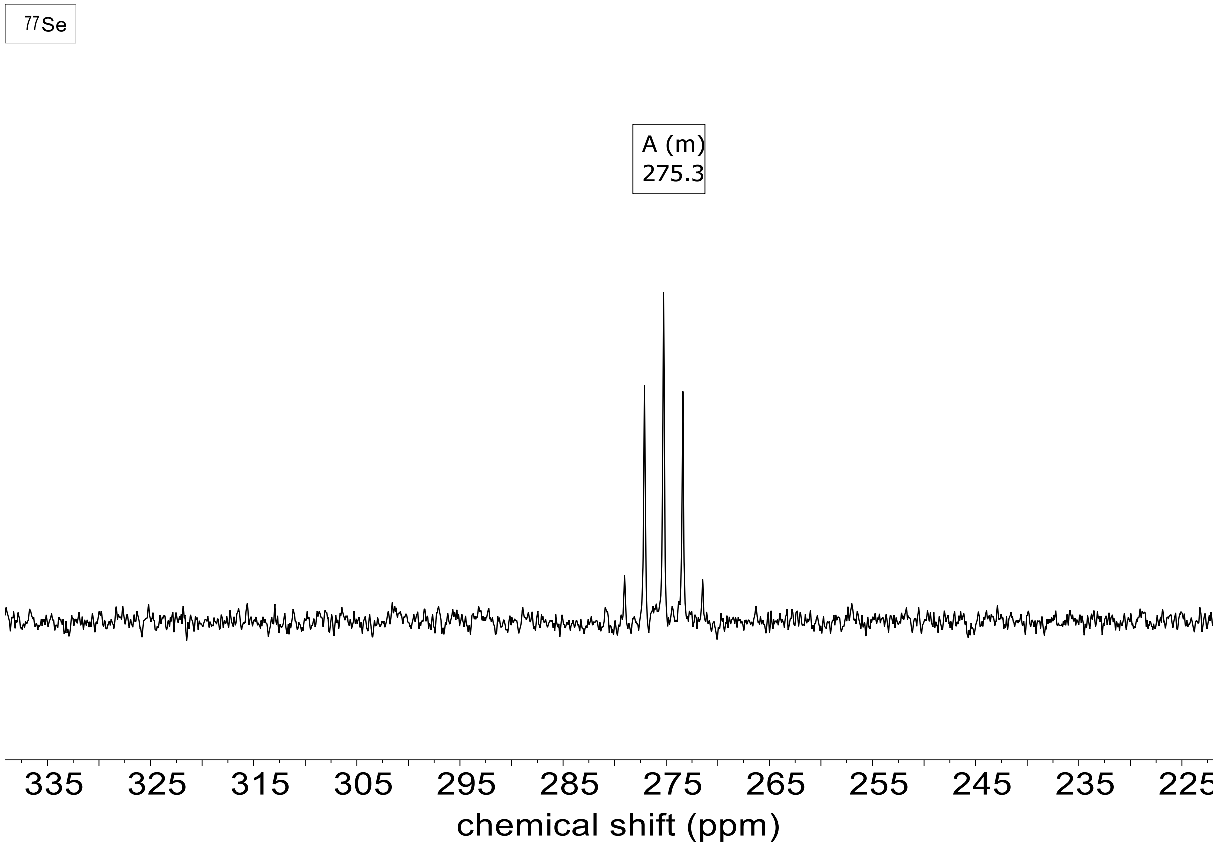
77Se
A (m)
275.3
335 325 315 305 295 285 275 265 255 245 235 225
chemical shift (ppm)
